# Identification and Comparative Expression Profiles of Chemoreception Genes Revealed from Major Chemoreception Organs of the Rice Leaf Folder, *Cnaphalocrocis medinalis* (Lepidoptera: Pyralidae)

**DOI:** 10.1371/journal.pone.0144267

**Published:** 2015-12-11

**Authors:** Fang-Fang Zeng, Zhen-Fei Zhao, Miao-Jun Yan, Wen Zhou, Zan Zhang, Aijun Zhang, Zhong-Xian Lu, Man-Qun Wang

**Affiliations:** 1 Hubei Insect Resources Utilization and Sustainable Pest Management Key Laboratory, College of Plant Science and Technology, Huazhong Agricultural University, Wuhan, 430070, P. R. China; 2 Invasive Insect Biocontrol and Behavior Laboratory, BARC-West, USDA-ARS, Beltsville, Maryland, 20705–2350, United States of America; 3 Institute of Plant Protection and Microbiology, Zhejiang Academy of Agricultural Sciences, Hangzhou, 310021, P. R. China; USDA-ARS, UNITED STATES

## Abstract

To better understand the olfactory mechanisms in the rice leaf folder, *Cnaphalocrocis medinalis* (Guenée), a serious pest of rice in Asia, we established six partial transcriptomes from antennae, protarsus, and reproductive organs of male and female adults. A total of 102 transcripts were identified, including 29 odorant receptors (ORs), 15 ionotropic receptors (IRs), 30 odorant-binding proteins (OBPs), 26 chemosensory proteins (CSPs), and 2 sensory neuron membrane proteins (SNMPs). The expression patterns of these genes were calculated by fragments per kilobase of exon per million fragments mapped (FPKM) and validated by real-time quantitative PCR (RT-qPCR). Some transcripts were exclusively expressed in specific organs, such as female protarsus, whereas others were universally expressed, this varied expression profile may provide insights into the specific functions mediated by chemoreception proteins in insects. To the best of our knowledge, among the 102 identified transcripts, 81 are novel and have never been reported before. In addition, it also is the first time that ORs and IRs are identified in *C*. *medinalis*. Our findings significantly enhance the currently limited understanding olfactory mechanisms of the olfactory mechanisms underlying the chemoreception system in *C*. *medinalis*.

## Introduction

The leaf folder, *Cnaphalocrocis medinalis* (Guenée) is a migratory rice pest in humid tropical and temperate regions of Oceania, Africa, and Asia [1]. The larvae can damage plants by folding the leaves and scraping the green leaf tissues within the fold, this activity reduces photosynthetic activity and causes yield losses [2]. Because of the cryptic feeding habits of larvae, chemical treatments are often impracticable. Chemical sensing mediates key behaviors in herbivorous insects, including seeking host plants, finding mating partners, selecting oviposition sites, and facilitating the detection of predators and toxic compounds [3]. Given this critical role in insect survival, regulating chemoreception as a means to control target insect pests is a potential safe and effective pest management measure.

Compared with vertebrates, insects have independent receptors localized in specific tissues, such as antennae, mouthparts, legs, wings, and ovipositor [4]. It is speculated that olfactory sensors mainly exist in antennae, whereas gustatory sensors can be found in legs [5], especially on the ventral surfaces of the tarsi (feet), where they come into contact with whatever they are walking on. Additionally, in some species, similar receptors are scattered over the surface of the body and may also be present on the egg-laying apparatus [4].

Chemosensation is orchestrated at various levels, starting with reception of semiochemicals at the periphery, processing of those signals at the antennal lobes, integration of olfactory and other sensory modalities in the higher processing centers of the brain, and ultimately translation of chemical signals into internal physiology and other external cues and signals [6]. During the peripheral process, diverse genes are utilized, including odorant binding proteins (OBPs), chemosensory proteins (CSPs), and chemosensory receptors [7, 8]. It is thought that external chemicals enter the chemosensilla at which point odorants or non-volatile chemicals are captured by odorant-binding proteins (OBPs) and/or chemosensory proteins (CSPs) [9], and then transported through the aquaeous sensillar lymph to the olfactory receptors. There the chemical messages are converted into electrical signals carrying information about the external world to the brain [10]. In addition, other chemosensory proteins have also been proposed to play a role in insect olfaction. Two critical classes are sensory neuron membrane proteins (SNMPs) [11, 12] and ionotropic receptors (IRs). OBPs and CSPs are both small water-soluble extra-cellular proteins containing a hydrophobic pocket [9, 13]. In Lepidoptera, most insect OBPs share six conserved cysteines (C1-X_25-30_-C2-X_3_-C3-X_36-42_-C4-X_8-14_-C5-X_8_-C6), whereas CSPs shares four (C1-X_6_-C2-X_18_-C3-X_2_-C4) [14]. It is now well accepted that OBPs and CSPs solubilize ligands, help transport hydrophobic molecules (towards odorants and pheromones) through the aqueous environment of the sensillar lymph, and contribute to the sensitivity of the insect olfactory system [15, 16]. However, besides this, OBPs and CSPs can also participate in physiological processes that extend beyond chemoreception [17, 18].

Chemosensory receptors (both olfactory and gustatory receptors) are trans-membrane proteins located in the dendrite membrane of receptor neurons. They play central roles as bio-transducers [7, 19]. The classical odorant receptors (ORs), similar to gustatory receptors (GRs), are extremely divergent seven-transmembrane domain (7TM) proteins, sharing as little as 8% amino acid identity within the same species [19]. In Drosophila, a novel OR sub-family, the odorant receptor co-receptor (Orco) was discovered, which is a single, atypical receptor that is co-expressed with conventional ORs in nearly all olfactory neurons [20]. Orco is highly conserved, sharing up to 94% sequence identity with orthologues amongst insect species [21]. It has been postulated that an olfactory receptor neuron (ORN) expresses one to three ligand-binding ORs with the conserved ubiquitous Orco [22]. The OR/Orco makes up a stand-alone heteromeric structure that functions as a ligand-gated ion channel to trigger the signal transduction cascade [21]. In Lepidopterans, numerous ORs specialized in the detection of sex pheromones, so-called pheromone receptors (PRs), have also been functionally characterized [23–27].

Recently, a new family of chemosensory receptors—the ionotropic receptor (IR) family was identified in *Drosophila melanogaster* by bioinformatic analyses [28]. IRs comprise a recently discovered ionotropic glutamate receptor (iGluR)—like protein family, involved in chemo-sensation [28]. Insect IRs contain structural regions that are conserved in iGluRs, including three trans-membrane domains, a two-way ligand-binding domain with two lobes, and one ion channel pore. However, the conserved iGluR glutamate-binding residues in the two lobes are not retained in IRs, indicating their atypical binding characters [28]. Unlike ORs, two or three IR genes appear to be always co-expressed with one or both of the conserved IR8a and IR25a genes in an IR-expressing neuron [29]. In *Drosophila*, IR64a and IR8a formed a functional ion channel that allowed ligand-evoked cation currents indicating that IR8a is a subunit that forms a functional olfactory receptor with IR64a in vivo to mediate odor detection [30]. Furthermore, IRs can be classified into two distinct subfamilies. One is the conserved ‘antennal IRs’, which have been proposed to be derived from an animal iGluR ancestor and thus represent the first olfactory receptor family in insects. The other is the species-specific ‘divergent IRs’, which have been implicated in taste and are derived from ‘antennal IR’ ancestors [31]. Additionally, sensory neuron membrane proteins (SNMPs) of which there are two sub-types of SNMP proteins, SNMP1 and SNMP2 in different insects [32], might also participate in chemoreception.

Identification of chemosensory genes is a prerequisite for functional characterization of olfactory genes. With the development of next generation sequencing (NGS) techniques, numerous chemoreception genes have been identified from various insect species, such as *Manduca sexta* [33], *Cydia pomonella* [26], *Aphis gossypii* [34], *Helicoverpa armigera* [35], *Sesamia inferens* [36], *Chilo suppressalis* [37], and *Spodoptera littoralis* [18, 38]. Although numerous chemosensory genes have been molecularly identified based on sequence similarity to reported genes in almost all insect orders, their exact functions are largely unknown. The expression profiles, particularly the tissue distribution, could provide important information on the functions of the chemosensory genes [39–41].

Previously, we reported sequencing and analyses of a *C*. *medinalis* antennal cDNA library and identified a subset of chemosensory genes corresponding to 5 OBPs and 3 CSPs [42]. Herein, to extend our view of the *C*. *medinalis* transcriptome and significantly increase the number of annotated olfactory genes, samples of the major olfactory organs, including antennae, protarsus, and reproductive organs from both male and female were analyzed. Our new analysis resulted in a total of 67,357 unigenes gene ontology (GO) annotations of which revealed enrichment in binding and catalytic activities. These data allowed us to identify novel *C*. *medinalis* olfactory genes, including binding proteins and chemosensory receptors. Furthermore, fragments per kilobase of exon per million fragments mapped (FPKM) calculations were performed to represent the expression levels, and real-time quantitative PCR (RT-qPCR) experiments were conducted on eight selected genes to validate our results.

## Materials and Methods

### Insect resources

*C*. *medinalis* larvae or pupae were collected from rice field in Wuxue, a county of Hubei Province in China (115°45′E; 30°00′N). The properties were not privately owned or protected in any way, and this field study did not involve endangered or protected species. The larvae were reared in buckets in the laboratory until pupation. Pupae were sexed and maintained separately inside glass tubes until moths emerged. Immediately after emergence, female and male adults were provided a 10% sucrose solution. To obtain mated females, newly emerged male and female moths were paired in plastic-screen cages (20 × 20 × 10 cm). Antennal (At), Protarsus (P), and reproductive organs (Ro) were isolated from mixed population adults 1–5 days after eclosion and kept separately in a freezer (- 80°C) until use.

### RNA preparation and cDNA library construction and sequencing

Frozen samples were individually crushed in a liquid nitrogen-cooled vitreous homogenizer and total RNA from each sample (~200 adult male or female moths) was extracted using TRIzol reagent (Invitrogen, Carlsbad, CA, USA) following the manufacturer’s instructions. Residual genomic DNA was removed using DNase I (Promega, Madison, WI, USA). Total RNA was dissolved in RNase-free water and RNA integrity was verified by gel electrophoresis. The RNA quantity was determined on a Nanodrop ND-2000 spectrophotometer (NanoDrop products, Wilmington, DE, USA).

Poly-A RNA was isolated from about 10–20 μg of the total RNA of different tissue samples was extracted using oligo (dT) magnetic beads. Then, poly-A RNA for each sample was digested into short fragments in a fragmentation buffer. Random hexamers were used for first-strand cDNA, followed by second-strand cDNA synthesis using RNase H and DNA polymerase I. These dual-strand DNA samples were treated with T4 DNA Polymerase and T4 Polynucleotide Kinase for end-repair and dA-tailing, followed by adaptor ligation to the dsDNA dA tail using T4 DNA ligase. Products corresponding to insert length ~200 bp were collected by 2% agarose gel electrophoresis and purified with a Takara quick Gel Extraction Kit (Takara, Hilden, Germany) and used as templates for PCR amplification to create a cDNA library. The library was pair-end sequenced using a PE90 strategy (paired-end reads of 90 base pairs per read) on an Illumina HiSeq^™^ 2000 (Illumina, San Diego, CA, USA) at the Beijing Genome Institute (Wuhan, China). Different libraries were sequenced in one lane; raw-reads were sorted by barcodes in the sequencing adaptor.

### Assembly and functional annotation

The clean-read dataset was generated from raw-reads by the following steps. First, reads with adaptors or those containing more than 5% unknown nucleotides (Ns) were directly removed. Second, low quality reads containing more than 20% suspect-nucleotides with a Phred Quality Score less than 10 were filtered out. Finally, both ends of the reads were evaluated to trim unreliable ends containing more than 3 successive suspect-nucleotides. Each clean-read dataset of all of the male and female tissues were separately de novo assembly using a paired-reads mode and default parameters in Trinity r2012-06-08 [43]. The Trinity outputs were clustered using TGICL [44]. Consensus cluster sequences and singletons made up the unigene dataset.

Those unigenes larger than 150 bp were first aligned using BLASTx to protein databases, including Nr, Nt, Swiss-Prot, KEGG, COG, and GO (e-value<1e-5). Proteins with the highest sequence similarity with the given unigenes along with their protein functional annotations were subsequently retrieved. BLAST results were then imported into Blast2GO pipeline [45] for GO Annotation. Protein coding region prediction was performed using OrfPredictor according to the BLAST results. Blast2GO was used to retrieve GO annotations of the unigenes with GO functional classification obtained using WEGO software. Then the best hit sequences were searched by BlastX in NCBI.

### Expression abundance analysis of unigenes

The expression abundance of the unigenes was calculated by the FPKM method [46] using the formula with software program: FPKM (A) = 106×C/ (N×L/103). In this formula, FPKM (A) is the expression abundance of unigene A; C is the number of fragments that uniquely aligned to gene A by SOAP [47]; N is the total number of fragments that uniquely aligned to all genes; and L is the total number of bases in gene A. The FPKM method can eliminate the influence of different gene lengths and sequencing discrepancy on the calculation of expression abundance. These results can be directly used to compare expression differences in samples.

### Phylogenetic analyses

The open reading frames (ORFs) of the putative chemosensory genes were predicted using ORF finder (http://www.ncbi.nlm.nih.gov/gorf/gorf.html). Putative N-terminal signal peptides of OBPs and CSPs were predicted using SignalP 4.1 Server (http://www.cbs.dtu.dk/services/SignalP/). The TMDs (Trans-Membrane Domains) of ORs and IRs were predicted using TMHMM Server Version 2.0 (http://www.cbs.dtu.dk/services/TMHMM). Amino acid sequence alignments were generated using WebLogo (http://weblogo.berkeley.edu/logo.cgi). Sequences were initially aligned using ClustalW and phylogenetic trees were constructed using the neighbor-joining method [48], with the Jones—Taylor—Thornton (JTT) amino acid substitution model as implemented in MEGA5.2 software (http://www.megasoftware.net/). Node support was assessed using a bootstrap procedure of 1000 replicates and uniform rates with pairwise deletion of data gaps.

### Quantitative real-time PCR validation

Total RNA was extracted from tissue samples according to the methods above. First-strand cDNA was synthesized according to the protocol provided with the One Step SYBR^®^ PrimeScript^®^ RT-PCR kit (Takara Code: DRR066A). The eight tested genes were selected and PCR primers were designed with the NCBI primer design tool Primer-BLAST (S1 Table). Tissue expression profiling of adults was carried out by a Bio-Rad IQ5 real-time qPCR system. Product sizes of ~100–160 bp were used to measure increased fluorescence of SYBR green. Real-time qPCR was conducted in 20 μl reactions that contained 10 μl 2× SYBR Green PCR Master Mix, 0.8 μl of each primer (10 mM), 2 μl sample cDNA, and 6.4 μl sterilized ultrapure H_2_O (Millipore). To properly assess the efficiency of PCR amplification, all the primers were tested by at least five orders of magnitude in multiples (5logs) consecutive dilutions of template concentration with at least 3 times parallel repeated. Then the efficiency can be controlled >90%. To assess reproducibility, test samples and endogenous controls were carried out in triplicate. Cycling parameters were as follows: 94°C for 2 min, 40 cycles at 95°C for 10 s, and 60°C for 30 s. Products were analyzed by agarose gel electrophoresis, sequencing, and melt curve analysis, which indicated that the respective reactions did not yield non-specific amplification products. CmedActin was used as an endogenous control to normalize the expression of target genes in olfactory tissues [42]. Female antennae of 0d old adults were used as a calibrator to calculate ΔΔCt values between tissues (ΔCt male antennae or female legs or other tissues at any time point– ΔCt female antennae of 0 d). Moreover, relative quantification was performed using the comparative 2^-ΔΔCt^ method [49] to identify differences in mRNA expression levels in different tissues and sexes of adults data indicate means ± SE (n = 3). One-way ANOVO statistical analysis was used to measure the different expression levels in tissues.

## Results and Discussion

### Assembly

Transcriptomic sequence data were generated using Illumina HiSeqTM2000/MiSeq technology. Approximately, 83.6, 90.2, 85.2, 84.5, 86.2, and 85.7 million raw-reads were obtained from libraries constructed from female and male antenna, tarsus, and reproductive organs, respectively. Additionally, 77.0, 83.3, 77.1, 76.7, 79.1, and 78.4 million respective clean-reads were obtained after filtering, followed by merging and clustering. A final transcript dataset with consisting of 67,357 unigenes was obtained. The dataset was 50.63 megabases with a mean length of 857 nt and an N50 of 1405 nt. There were 18,943 unigenes longer than 1000 nt, which accounted for 28.12% of the transcriptome assembly (Table 1).

**Table 1:** Data summary.

	Sample	Counts(total nb)	Total length	Mean length	N50	Consensus Sequeces	Distinct Clusters	Distinct Singletons
	Female Antennae	135,749	38,145,858	281	389	_	_	_
	Female ovipositorFe	140,371	36,913,097	263	354	_	_	_
	Male tarsus	121,628	32,420,687	267	357	_	_	_
Contig	Male antennae	120,037	42,510,286	354	627	_	_	_
	Male reproductive organs	126,108	34,488,011	273	364	_	_	_
	Male tarsus	127457	32,729,371	257	329	_	_	_
	Female Antennae	73,996	35,682,190	482	656	73,996	11,590	62,406
	Female ovipositor	69,100	33,135,277	480	696	69,100	12,464	56,636
	Female tarsus	62,338	28,960,889	465	654	62,338	9,778	52,560
Unigene	Male antennae	71,058	43,759,881	616	987	71,058	12,412	57,646
	Male reproductive organs	68,047	31,559,480	464	618	68,047	10,184	57,863
	Male tarsus	66827	29,066,891	435	548	66,827	9,189	57,638
	Merge	67,357	57,697,863	857	1405	67,357	21,666	45,691

### Homology analysis and gene ontology annotation

**Fig 1:**
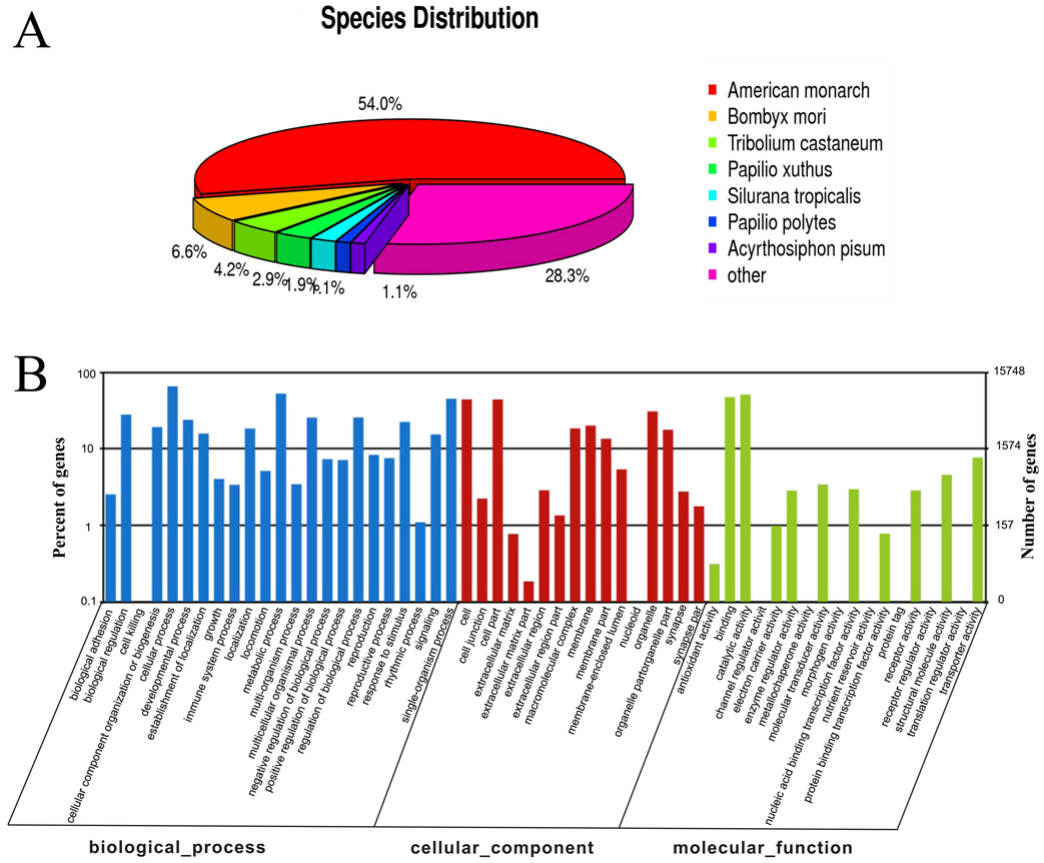
Annotation summary of *C*. *medinalis* antenna unigenes. (A) Species distribution of unigenes’ best-hit annotation term in the nr database (B) Gene ontology classifications of the *C*. *medinalis* unigenes.

Among the 67,357 unigenes, 36,966 (54.8%) were matched using a BLASTX homology search to entries in the NCBI non-redundant (nr) protein database with a cut-off E-value of 10–5. The highest percentage of matched sequences (refers to only the best match for each unigene to a sequence in the blast database) was to *Danaus plexippus* (54.0%), followed by *Bombyx mori* (6.6%), *Tribolium castaneum* (4.2%), *Papilio xuthus* (2.9%), *Silurana tropicalis* (1.9%), *Papilio polytes* (1.1%), and *Acyrthosiphon pisum* (1.1%). The remaining 28.3% sequences were matched to other insect species (Fig 1A).

Gene Ontology (GO) annotations were used to classify the 67,357 unigenes into different functional groups by BLAST2GO. Based on sequence homology, 15,748 unigenes (23.37%) could be annotated with each unigene classified into one or more functional groups of the three biological processes (Fig 1B). In the molecular function category, genes expressed in the antennae were mostly enriched in molecular binding activity (e.g., nucleotide, ion, and odorant binding) and catalytic activity (e.g., hydrolase and oxidoreductase). Among the biological process terms, cellular and metabolic processes were the most frequently represented, and in the cellular component terms, cell, cell part, and organelle were the most abundant (Fig 1B). These results are comparable to the reported *Chilo suppressalis* transcriptional profile [37].

### Identification of chemosensory receptor candidates

The unigenes related to chemosensory receptor candidates were identified by keyword searches of BLASTx annotations. To identify additional OR candidates, the predicted protein sequences of the unigenes were further searched by PSI-blastp with known lepidopteran chemosensory receptors (Tables 2 and 3). We identified 29 distinct unigenes that were candidates for OR genes and no GR gene like sequences longer than 200bp were identified in any tissue even tarsus. Among the OR genes, four sequences were full length ORs because they had an intact open reading frame with a general length of 1200 bp and 4 to 7 predicted transmembrane domains, which are characteristic of typical insect ORs. Based on typical OR lengths (around 400 amino acids), over 60% of the sequences identified represented fragments with missing 5’ ends and only seven contained a deduced protein longer than 200 amino acids. Similar results have been reported in the transcriptomes of other species, such as *S*. *Inferens* [36], *C*. *suppressalis*, and *H*. *armigera*.

**Table 2:** Unigenes of candidates for olfactory receptors.

Gene name	Accession number	Unigene reference	ORF(aa)	Status	TMD	Evalue	Blastx best hit
CmedPR1	KP975162	Cl195	375	complete	4	2e-76	gb|AGK43827.1| odorant receptor 5 [*Plutella xylostella*]
CmedPR2	KP975163	Cl1885	434	complete	6	6e-95	gb|ADB89183.1| odorant receptor 6 [*Ostrinia nubilalis*]
CmedPR3	KP97514	Unigene2572	290	5’, 3’ missing	2	3e-76	gb|AGG91650.1| odorant receptor [*Ostrinia furnacalis*]
CmedPR4	KP975165	unigene7146	159	5’ missing	2	3e-18	dbj|BAI66624.1| odorant receptor [*Ostrinia nubilalis*]
CmedOrco	KP975160	Unigene8047	473	complete	7	0.0	gb|AGS41440.1|Odorant receptor co-receptor [*Agrotis segetum*]
CmedOR1	KP975136	Unigene5798	448	complete	6	0.0	gb|AIT72007.1| olfactory receptor 47, partial [*Ctenopseustis obliquana*]
CmedOR2	KP975137	Unigene8211	407	complete	7	3e-101	NP_001091792.1Candidate olfactory receptor[*Bombyx mori*]
CmedOR3	KP975138	Cl4164	230	3’ missing	4	1e-81	gb|AIG51891.1| odorant receptor, partial [*Helicoverpa armigera*]
CmedOR4	KP975139	Unigene33571	216	5’, 3’ missing	3	9e-75	gb|AFL70813.1| odorant receptor 50, partial [Manduca sexta]
CmedOR5	KP975140	unigene2989	214	5’ missing	4	1e-62	NP_001157210.1| olfactory receptor 17 [*Bombyx mori*]
CmedOR6	KP975141	unigene14729	211	3’ missing	3	1e-24	gb|AII01081.1| odorant receptors [*Dendrolimus kikuchii*]
CmedOR7	KP975142	cl1990	207	5’ missing	4	5e-88	gb|AIG51891.1| odorant receptor, partial [*Helicoverpa armigera*]
CmedOR8	KP975143	cl7710	201	5’ missing	3	4e-70	gb|AII01102.1| odorant receptors [*Dendrolimus kikuchii*]
CmedOR9	KP975144	cl282	192	5’ missing	1	7e-52	gb|AFL70813.1| odorant receptor 50, partial [*Manduca sexta*]
CmedOR10	KP975145	unigene11382	179	5’ missing	2	6e-97	|gb|AIG51892.1| odorant receptor [*Helicoverpa armigera*]
CmedOR11	KP975146	unigene7039	172	5’ missing	2	2e-67	gb|AIG51899.1| odorant receptor [*Helicoverpa armigera*]
CmedOR12	KP975147	unigene20786	169	5’ missing	2	2e-75	gb|AIG51873.1| odorant receptor [*Helicoverpa armigera*]
CmedOR13	KP975148	unigene30983	167	5’ missing	2	2e-75	gb|ACM18061.1| putative odorant receptor OR3 [*Manduca sexta*]
CmedOR14	KP975149	unigene35520	164	5’, 3’ missing	1	2e-76	gb|AII01061.1| odorant receptors [*Dendrolimus houi*]
CmedOR15	KP975150	unigene14982	163	5’, 3’ missing	2	2e-11	gb|AIT72018.1| olfactory receptor 67 [*Ctenopseustis obliquana*]
CmedOR16	KP975151	unigene17854	163	5’ missing	2	2e-66	gb|AIG51883.1| odorant receptor, partial [*Helicoverpa armigera*]
CmedOR17	KP975152	unigene14624	162	5’, 3’ missing	3	3e-46	gb|AIT71986.1| olfactory receptor 12 [*Ctenopseustis obliquana*]
CmedOR18	KP975153	unigene3174	150	5’, 3’ missing	3	1e-44	gb|AIT69895.1| olfactory receptor 46, partial [*Ctenopseustis herana*]
CmedOR19	KP975154	unigene18118	139	5’, 3’ missing	2	8e-67	gb|AFC91721.1| putative odorant receptor OR12 [*Cydia pomonella*]
CmedOR20	KP975155	unigene24580	118	5’, 3’ missing	2	5e-67	gi|669092426|gb|AII01085.1| odorant receptors [*Dendrolimus kikuchii*]
CmedOR21	KP975156	unigene7667	118	5’ missing	1	1e-58	gb|AIG51873.1| odorant receptor [*Helicoverpa armigera*]

**Table 3:** Unigenes of candidates for olfactory receptors.

Gene name	Accession number	Unigene reference	ORF(aa)	Status	TMD	Evalue	Blastx best hit
CmedOR22	KP975157	unigene2843	115	5’ missing	1	4e-35	gb|AII01110.1| odorant receptors [*Dendrolimus kikuchii*]
CmedOR23	KP975158	unigene18065	99	3’ missing	1	4e-26	gb|AIT69911.1| olfactory receptor 71 [*Ctenopseustis herana*]
CmedOR24	KP975159	unigene35280	84	5’ 3’ missing	1	7e-40	gb|AIG51878.1| odorant receptor, partial [*Helicoverpa armigera*]

**Fig 2:**
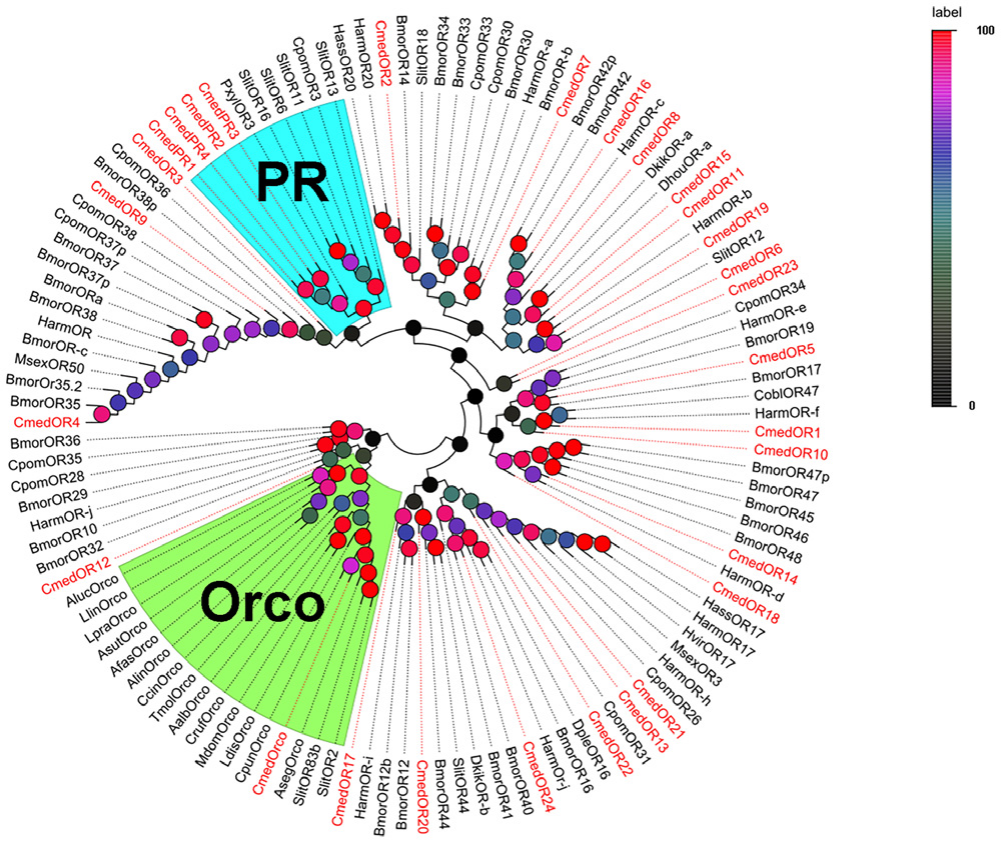
Phylogenetic tree of candidate CmedORs with known OR sequences of other species. Harm: *H*. *armigera*; Hvir: *H*. *virescens*; Bmor: *B*. *mori*; Pxyl: *P*. *xylostella*; Cpom: *Cydia pomonella*, Hass: *H*. *assulta*; Msex: *Manduca sexta*, Slit: *Spodoptera littoralis*; Dkik: *Dendrolimus kikuchii*; Dhou: *Dendrolimus houi*; Cobl: *Ctenopseustis obliquana*; Dple: *Danaus plexippus*; Alin: *Adelphocoris lineolatus*; Afas: *Adelphocoris fasciaticollis*; Asut: *Adelphocoris suturalis*; Lpra: *Lygus pratensis*; Llin: *Lygus lineolaris*; Aluc: *Apolygus lucorum*; Tmol: *Tenebrio molitor*; Ccin: *Cephus cinctus*; Mdom: *Musca domestica*; Cruf: *Chrysomya rufifacies*; Aalb: *Aedes albopictus*; Ldis: *Lymantria disparasiatica*; Cpun: *Conogethes punctiferalis*; Aseg: *Agrotis segetum*; The clades in blue and in green indicate the pheromone receptor gene clade and the co-receptor gene clade respectively.

In the phylogenetic analyses, lepidopteran putative pheromone receptors clustered in a subgroup (Fig 2). These four OR candidates were named “CmedPRx” (x = 1 through 4) to more clearly indicate putative function; this nomenclature was adopted to more clearly differentiate lepidopteran pheromone receptors from general odorant receptors, which generally have been poorly classified. The *C*. *medinalis* Orco orthologue, termed-CmedOrco, had a high degree of identity with other insect co-receptors. Almost all CmedOR candidates clustered with at least one putative lepidopteran orthologous in the phylogenetic tree.

Compared to genome-based identification the number of ORs identified in transcriptome analyses is typically limited. The number of ORs identified in the genomes of *D*. *melanogaster*, *A*. *gambiae*, and *B*. *mori* are 62, 79 and 72, respectively, while in antennal transcriptomes 47 were identified in *M*. *sexta* [33], 43 in *C*. *Pomonella* [26] and 47 in *H*. *armigera* [35]. In the European corn borer (*Ostrinia nubilalis*), a neuroanatomical study suggested 64 glomeruli in the antennal lobe of both genders [19], and in *H*. *virescens*, the total number of glomeruli, including dimorphic and isomorphic units, consisted of 64 in males and 62 in female [50]. It is believed that that one olfactory receptor type is expressed in the OSN and axonal projects of different OSNs express the same olfactory receptor, which converges on the same antennal lobe glomeruli. Indeed, some glomeruli can also be activated by OSNs that express other classes of chemoreceptors, such as ionotropic receptors [29]. Although there has been no reports on the number of glomeruli in *C*. *medinalis*, our dataset of 29 OR sequences is somewhat smaller than those of other insects [36, 37] even though the sequencing depth of our transcriptome was greater than others. There are several possible explanations to address this potential discrepancy. First, according to the sequence reads, the expression level of ORs in the *C*. *medinalis* antenna is very low, suggesting low transcript numbers (S1 Fig), resulting in lower detection metrics, which suggests that there is a high chance to identify additional low expression *C*. *medinalis* chemosensory genes that were missed in our assembly. Second, it is possible that the remaining OR and GR genes are either exclusively expressed in other olfactory organs such as maxillary palp (especially OR) or are temporally restricted to the developmental period (embryonic, larval, or pupal).

### Identification of candidates for sensory neuron membrane proteins and ionotropic receptors

Sensory neuron membrane proteins (SNMPs), which are located in the dendritic membrane of primarily pheromone-specific OSNs, are thought to trigger ligand delivery to the receptor [32]. The two ‘discovered’ SNMPs share 100% sequence similarity with those reported previously [51].

**Fig 3:**
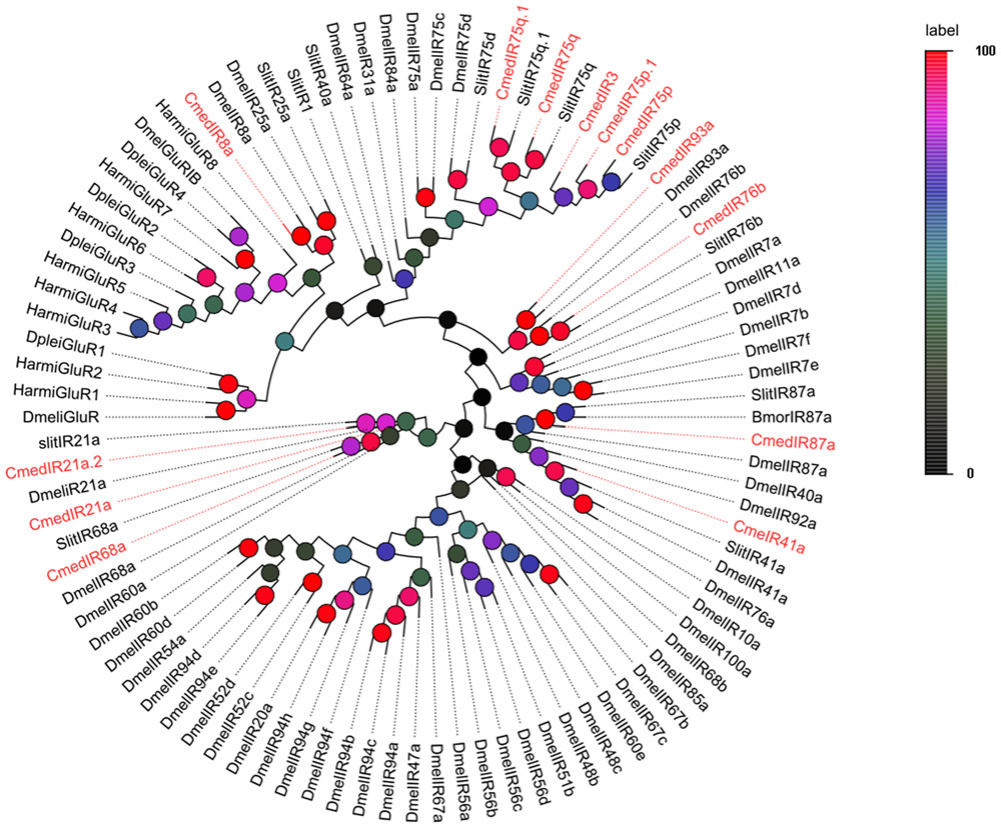
Phylogenetic tree of candidate CmedIRs with known IR sequences of other species. Harm: *H*. *armigera*; Hvir: Bmor: *B*. *mori*; Slit: *Spodoptera littoralis*, Dmel: *Drosophila melanogaster*.

The putative IR genes in the *C*. *medinalis* antennal transcriptome can be represented according to their similarity to known insect IRs. Bioinformatics analysis led to the identification of 15 IR candidates, in which three sequences contained a full-length ORF, and the remaining 12 sequences were fragments. Insect IRs have three trans-membrane domains, and TMHMM2.0 predicted the six candidates *C*. *medinalis* IR also have three trans-membrane domains (Table 4). For phylogenetic analysis, 13 of the putative *C*. *medinalis* IRs were aligned with orthologous IRs from *D*. *plexippus*, *B*. *mori*, *S*. *Littoralis*, and *D*. *melanogaster*, the remaining two *C*. *medinalis* IR sequences were too short to align successfully. From the phylogenetic tree, we detected clear segregation between the different subfamilies, such as iGluR, IR75q, IR41a, and IR93a. In this phylogenetic tree, the most of *C*. *medinalis* IR candidates clustered with orthologous ionotropic receptors into separate clades (Fig 3). Based on their positions in the phylogenetic tree and strong bootstrap support, 12 of the 13 analyzed *C*. *medinalis* IRs candidates clustered with the IR8a, IR75p, IR75q, IR21a, IR41a, IR68a, IR76b, IR87a, and IR93a groups, which formed small expansions with other putative genes. Similar to previous reports [28, 31], IRs and iGluRs clustered phylogenetically into separate clades, with the exception of the IR8a and IR25a lineages, which clustered with the iGluRs. Unexpectedly, an orthologe of co-receptor IR25a, which is typically among the highest expressed IR transcripts in other insect antennae, was not found in our transcriptome.

**Table 4:** Unigenes of candidates for ionotropic receptors.

Gene name	Accession number	Unigene reference	ORF(aa)	Status	TMD	Evalue	Blastx best hit
CmedIR93a	KP975113	Cl8524	874	Complete	4	0.0	XP_004925511.1 glutamate receptor 2-like [*Bombyx mori*]
CmedIR8a	KP975100	Unigene29958	849	5’ missing	3	0.0	gb|AII01121.1| ionotropic receptors [*Dendrolimus kikuchii*]
CmedIR76b	KP975099	Cl4160	552	Complete	3	0.0	XP_004927780.1 glutamate receptor ionotropic, delta-2-like isoform X1 [*Bombyx mori*]
CmedIR75p.2	KP975109	Cl8802	465	5’, 3’missing	3	0.0	|gb|ADR64684.1| putative chemosensory ionotropic receptor IR75p [*Spodoptera littoralis*]
CmedIR75q.2	KP975110	Unigene842	434	5’, 3’ missing	3	0.0	gb|AFC91752.1| putative ionotropic receptor IR75q2 [*Cydia pomonella*]
CmedIR87a	KP975112	Unigene11123	430	Complete	3	0.0	gb|AFC91760.1| putative ionotropic glutamate receptor 87a, partial [*Cydia pomonella*]
CmedIR75q.1	KP975108	Cl4222	400	5’ lost	3	2e-117	|gb|ADR64685.1| putative chemosensory ionotropic receptor IR75.2 [*Spodoptera littoralis*]
CmedIR21a.1	KP975104	Unigene15010	362	5’ lost	2	4e-154	gb|AII01123.1| ionotropic receptors [*Dendrolimus kikuchii*]
CmedIR75p	KP975101	Cl5125	324	5’, 3’ missing	1	4e-119	gb|AII01128.1| ionotropic receptors [*Dendrolimus kikuchii*]
CmedIR21a.2	KP975105	Unigene34047	317	5’, 3’ missing	1	0.0	gb|AII01123.1| ionotropic receptors [*Dendrolimus kikuchii*]
CmedIR1	KP975111	Unigene843	188	5’, 3’ missing	1	3e-109	gb|AII01119.1| ionotropic receptors [*Dendrolimus houi*]
CmedIR68a	KP975107	Unigene21319	149	5’, 3’ missing	1	6e-70	gb|AIG51921.1| ionotropic receptor, partial [*Helicoverpa armigera*]
CmedIR3	KP975102	Unigene11279	114	5’, 3’ missing	1	2e-40	gb|AII01118.1| ionotropic receptors, partial [*Dendrolimus houi*]
CmedIR41a	KP975106	Unigene42566	80	5’, 3’ missing	1	9e-33	gb|ADR64681.1| putative chemosensory ionotropic receptor IR41a [*Spodoptera littoralis*]
CmedIR4	KP975103	Unigene18142	78	5’missing	1	3e-37	gb|AIG51921.1| ionotropic receptor, partial [*Helicoverpa armigera*]
CmedSNMP1	AFG73002	Cl7052	525	complete	2	0.0	gi|383215100|gb|AFG73002.1| sensory neuron membrane protein 1 [*Cnaphalocrocis medinalis*]
CmedSNMP2	AFG73003	Unigene4172	520	complete	1	0.0	gi|383215102|gb|AFG73003.1| sensory neuron membrane protein 2 [*Cnaphalocrocis medinalis*]

This is the first report of IRs in *C*. *medinalis* and sequence alignments showed that the IRs are more highly conserved across species and orders than the ORs are. In our database, we identified fragments of putative IRs, which were specifically and highly expressed in the antennae of both sexes. Data for these genes, including unigene reference numbers, length, and best BLASTx hit for all the 15 IRs, are listed in Table 4. The sequences of all 15 IRs are listed in S1 File.

### Identification of putative odorant-binding proteins and chemosensory proteins

**Fig 4:**
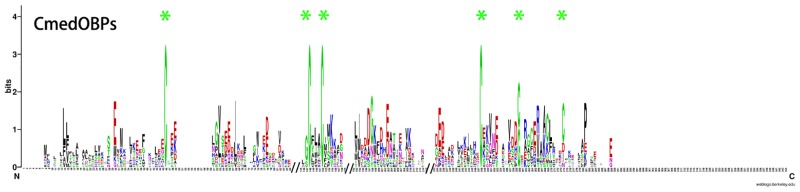
CmedOBPs sequence logo. Degree of amino acid sequence conservation along the primary sequence axis of *C*. *medinalis* odorant-binding proteins (OBPs). Depicted amino acid character size correlates to relative conservation across aligned sequences. Green asterisks indicate the conserved six cysteine motifs characteristic of OBPs.

**Fig 5:**
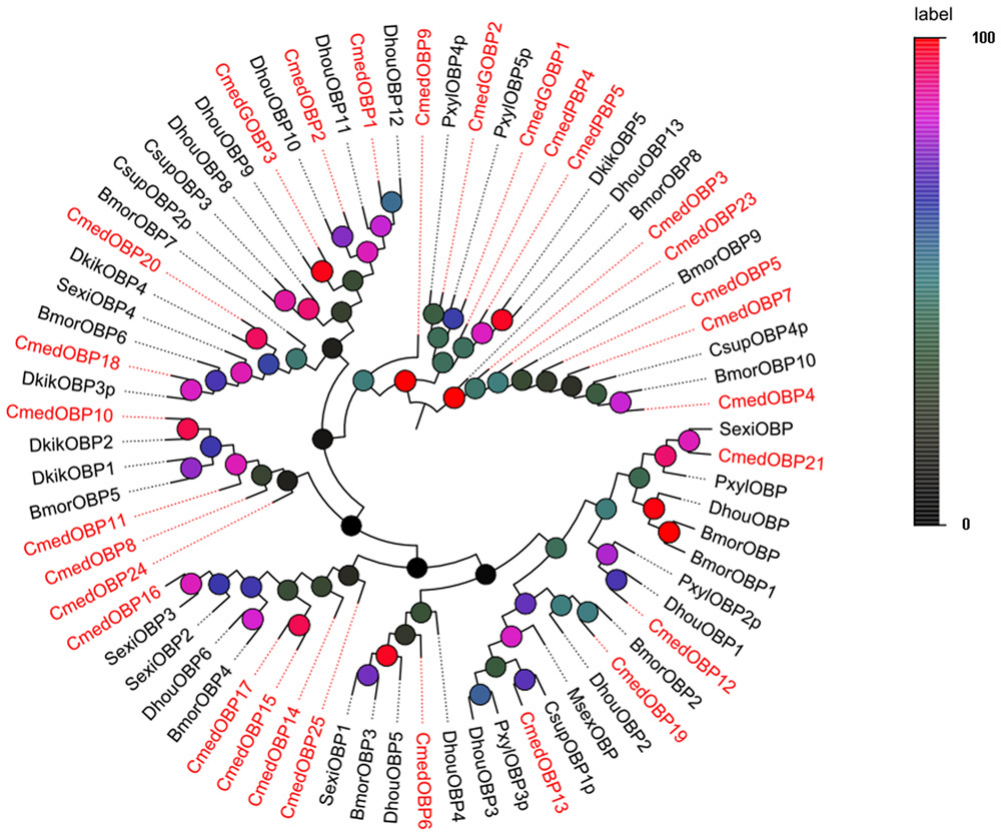
Phylogenetic tree of candidate CmedOBPs with known OBP sequences of other species. Harm: *H*. *armigera*; Hvir: *H*. *virescens*; Bmor: *B*. *mori*; Pxyl: *P*. *xylostella*; Hass: *H*. *assulta*; Msex: *Manduca sexta*, Sexi: *Spodoptera exigua*; Slit: *Spodoptera littoralis*; Dkik: *Dendrolimus kikuchii*; Dhou: *Dendrolimus houi*; Dple: *Danaus plexippus*; Csup: *Chilo suppressalis*.

In addition to a keyword searching and PSI-Blast, we also used a motif scan for the conserved 6 cysteine residue pattern (C1-X_5-39_-C2-X_3_-C3-X_21-44_-C4-X_7-12_-C5-X_8_-C6) characteristic of odorant-binding proteins [52]. In our transcriptome data set, we identified 30 sequences that potentially encode odorant-binding proteins, including six previously annotated OBPs. Among these 30 sequences, 22 had an intact ORF detected, six unigenes lacked signal peptides and the other two were missing the 5’ sequence. Sequence alignment showed that 15 of the 22 putative intact OBPs had the classic cysteine motif (Fig 4). The number of CmedOBPs candidate identified was far less than the 46 annotated OBPs found in the *B*. *mori* genome [53], but was more than the 18 putative OBPs identified in *M*. *sexta* [33]. In the phylogenetic tree, the PBP and GOBP sequences were clustered into the PBP and GOBP clades, respectively, as expected (Fig 5). All candidates for OBP sequences were clustered with at least one lepidopteran ortholog. By comparing our putative OBPs with the NCBI records for *C*. *medinalis*, we identified six previously annotated “genes”, GOBP1, GOBP2, GOBP3, PBP4, OBP1, and OBP2. All of the previously annotated sequences had >99% amino acid identity with their most similar NCBI records. Therefore, we named these candidates GOBPs and PBPs based on their existing NCBI records, and named the other OBP candidates “CmedOBP” followed by a number in descending order of their coding lengths (Tables 5 and 6).

**Table 5:** Unigenes of candidates for odorant binding proteins.

Gene name	Accession number	Unigene reference	ORF(aa)	status	Signal peptide	E-value	BLASTx best hit
CmedGOBP1	AFG72996	Cl3280.coontig1	163	complete	19	5e-102	gi|383215088|gb|AFG72996.1| general odorant binding protein 1 [*Cnaphalocrocis medinalis*]
CmedGOBP2	KC507183	Cl9173.contig3	161	complete	22	1e-108	gi|472271932|gb|AGI37366.1| general odorant-binding protein 2 [*Cnaphalocrocis medinalis*]
CmedGOBP3	KC507179	Unigene23178	173	complete	N	1e-97	gi|472271924|gb|AGI37362.1| general odorant-binding protein 3 [*Cnaphalocrocis medinalis*]
CmedPBP4	KC507185	Unigene4439	163	complete	N	3e-101	gi|472271936|gb|AGI37368.1| pheromone binding protein 4 [*Cnaphalocrocis medinalis*]
CmedPBP5	KP975161	Unigene25298	169	complete	26	1e-53	gb|ACX47891.1| pheromone-binding protein 2 F102 precursor [*Amyelois transitella*]
CmedOBP1	AFG72998	Unigene26852	147	complete	26	5e-99	gi|383215092|gb|AFG72998.1| odorant-binding protein 1 [*Cnaphalocrocis medinalis*]
CmedOBP2	AFG73000	Unigene33027	129	complete	18	1e-93	gi|383215096|gb|AFG73000.1| odorant-binding protein 2 [*Cnaphalocrocis medinalis*]
CmedOBP3	KP975114	Unigene2099	256	complete	N	1e-74	ref|NP_001153663.1| odorant binding protein LOC100301495 precursor [*Bombyx mori*]
CmedOBP4	KP975115	Unigene4118	254	complete	N	4e-134	gb|ADD71058.1| odorant-binding protein [*Chilo suppressalis*]
CmedOBP5	KP975116	Unigene29539	240	complete	18	5e-77	ref|NP_001157372.1| odorant binding protein fmxg18C17 precursor [*Bombyx mori*]
CmedOBP6	KP975117	Unigene19679	231	complete	24	8e-75	|gb|AII00994.1| odorant binding protein [*Dendrolimus kikuchii*]
CmedOBP7	KP975118	Cl1968	223	5’, 3’ missing	—	2e-68	gb|EHJ70925.1| odorant binding protein fmxg18C17 [*Danaus plexippus*]
CmedOBP8	KP975119	Unigene44356	219	complete	17	8e-70	gb|AIL54057.1| odorant-binding protein 21, partial [*Chilo suppressalis*]
CmedOBP9	KP975120	Unigene44227	204	5’ missing	—	3e-111	gb|AGK24579.1| odorant-binding protein 3 [*Chilo suppressalis*]
CmedOBP10	KP975121	Unigene6269	187	complete	20	5e-51	gb|AII01008.1| odorant binding protein [*Dendrolimus kikuchii*]
CmedOBP11	KP975122	Cl8457	171	complete	N	7e-72	gb|AGK24580.1| odorant-binding protein 4 [*Chilo suppressalis*]
CmedOBP12	KP975123	Cl3709	158	5’, 3’ missing	—	6e-38	ref|NP_001140188.1| odorant-binding protein 4 [*Bombyx mori*]
CmedOBP13	KP975124	Cl4036	152	complete	22	1e-63	gb|AER27567.1| odorant binding protein [*Chilo suppressalis*]
CmedOBP14	KP975125	Unigene36228	149	complete	25	2e-08	gb|AEB54582.1| OBP3 [*Helicoverpa armigera*]
CmedOBP15	KP975126	Unigene22885	148	complete	20	2e-15	|gb|AAR28762.1| odorant-binding protein [*Spodoptera frugiperda*]
CmedOBP16	KP975127	Unigene11126	146	complete	19	6e-50	gb|AGH70102.1| odorant binding protein 6 [*Spodoptera exigua*]
CmedOBP17	KP975128	Unigene33680	140	complete	16	6e-08	gb|AFD34177.1| odorant binding protein 1 [*Argyresthia conjugella*]
CmedOBP18	KP975129	Unigene33154	137	complete	18	2e-48	gb|AII00979.1| odorant binding protein [*Dendrolimus houi*]
CmedOBP19	KP975130	Unigene34044	130	3’ missing	20	4e-26	gb|AGP03455.1| SexiOBP9 [*Spodoptera exigua*]

**Table 6:** Unigenes of candidates for odorant binding proteins.

Gene name	Accession number	Unigene reference	ORF(aa)	status	Signal peptide	E-value	BLASTx best hit
CmedOBP20	KP975131	Cl2821	123	complete	16	9e-74	gb|AGK24578.1| odorant-binding protein 2 [*Chilo suppressalis*]
CmedOBP21	KP975132	Unigene11641	114	complete	N	1e-62	ref|XP_004928230.1| PREDICTED: pheromone-binding protein-related protein 2-like [*Bombyx mori*]
CmedOBP22	KP975133	Unigene24577	110	5’, 3’ missing	—	9e-24	|ref|NP_001157372.1| odorant binding protein fmxg18C17 precursor [*Bombyx mori*]
CmedOBP23	KP975134	Unigene36708	106	5’ missing	—	4e-21	ref|NP_001157372.1| odorant binding protein fmxg18C17 precursor [*Bombyx mori*]
CmedOBP24	KP975135	Unigene43762	99	5’ missing	—	3e-57	gb|EHJ74351.1| odorant-binding protein 2 [*Danaus plexippus*]
CmedOBP25	KP987795	Unigene37903	93	5’ missing	—	4e-06	gb|AGC92789.1| odorant-binding protein 9 [*Helicoverpa assulta*]

**Fig 6:**
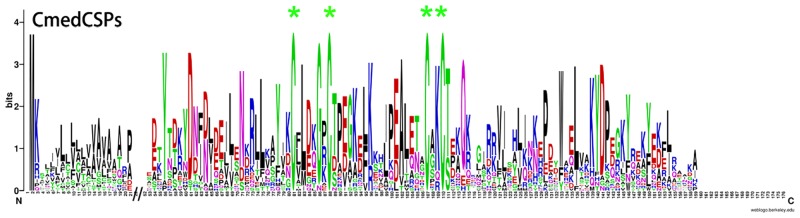
CmedCSPs sequence logo. Degree of amino acid sequence conservation along the primary sequence axis of *C*. *medinalis* chemosensory proteins (CSPs). Depicted amino acid character size correlates to relative conservation across aligned sequences. Green asterisks indicate the conserved four cysteine motifs characteristic of CSPs.

**Fig 7:**
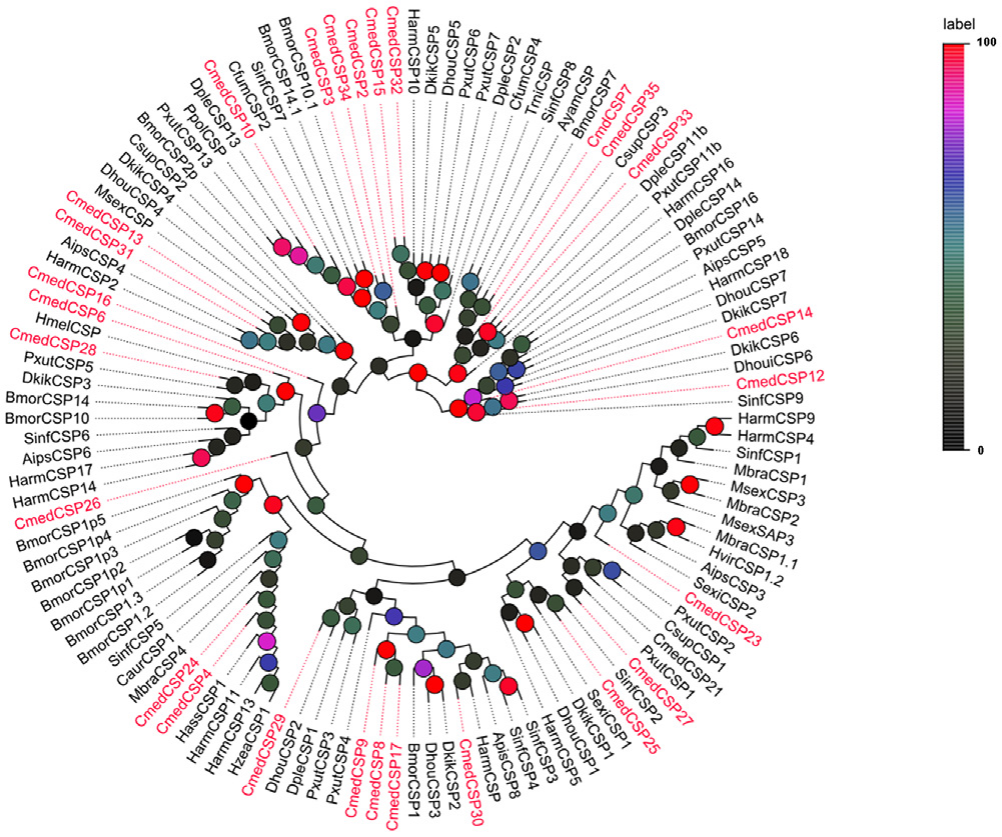
Phylogenetic tree of candidater CmedCSPs with known CSP sequences of other species. Harm: *H*. *armigera*; Hvir: *H*. *virescens*; Bmor: *B*. *mori*; Pxyl: *P*. *xylostella*; Hass: *H*. *assulta*; Hzea: *Helicoverpa zea*; Msex: *Manduca sexta*, Mbra: *Mamestra brassicae*; Slit: *Spodoptera littoralis*; Sexi: *Spodoptera exigua*; Dkik: *Dendrolimus kikuchii*; Dhou: *Dendrolimus houi*; Dple: *Danaus plexippus*; Apis: *Agrotis ipsilon*; Sinf: *Sesamia inferens*; Csup: *Chilo suppressalis*; Caur: *Chilo auricilius*; Cfum: *Choristoneura fumiferana*; Trni: *Trichoplusia ni*; Ayam: *Antheraea yamamai*; Ppol: *Papilio polytes*.

Bioinformatics analysis led to the identification of 26 sequences that encoded CSPs candidates (Fig 6). Among them, 20 sequences had full-length ORFs and signal peptides, which were found by using the SignalP test, except for CmedCSP15. Neighbor-joining tree analysis showed that all 26 sequences clustered with orthologous Lepidopteran genes (Fig 7). These CSP candidates were named “CmedCSPx” followed by a number in descending order of the length of the coding region. Information of the CSPs is presented in Table 7. The CSP sequences are listed in S1 File.

**Table 7:** Unigenes of candidates for chemosensory proteins.

Gene name	Accession number	Unigene reference	ORF(aa)	Status	Signal peptide	E-value	BLASTx best hit
CmedCSP2	KC507180	Unigene26762	124aa	complete	16	6e-72	gi|472271926|gb|AGI37363.1| chemosensory protein 2 [*Cnaphalocrocis medinalis*]
CmedCSP3	KC507182	Cl2512.contig2	123aa	complete	17	4e-66	gi|472271930|gb|AGI37365.1| chemosensory protein 3 [*Cnaphalocrocis medinalis*]
CmedCSP4	KM365188	Unigene4140	131aa	complete	18	5e-75	gi|723592548|gb|AIX97823.1chemosensory protein [*Cnaphalocrocis medinalis*]
CmedCSP6	KM365190	Unigene16713	129aa	complete	18	1e-80	gi|723592548|gb|AIX97825.1chemosensory protein [*Cnaphalocrocis medinalis*]
CmedCSP7	KM365191	Unigene29759	123aa	complete	19	6e-69	gi|723592548|gb|AIX97826.1chemosensory protein [*Cnaphalocrocis medinalis*]
CmedCSP10	KM365194	Unigene7213	121aa	complete	17	2e-75	gi|723592548|gb|AIX97829.1chemosensory protein [*Cnaphalocrocis medinalis*]
CmedCSP12	KM365196	Unigene11453	105aa	complete	18	5e-54	gi|723592548|gb|AIX97831.1chemosensory protein [*Cnaphalocrocis medinalis*]
CmedCSP13	KM365197	Cl270.coontig1	120aa	complete	16	3e-60	gi|723592548|gb|AIX97832.1| chemosensory protein [*Cnaphalocrocis medinalis*]
CmedCSP15	AIX97837	Unigene22875	120aa	complete	No	2e-72	gi|723592548|gb|AIX97837.1chemosensory protein [*Cnaphalocrocis medinalis*]
CmedCSP14	KM365198	CL1055.Contig1	108aa	complete	18	2e-47	gi|669092282|gb|AII01013.1| chemosensory proteins [*Dendrolimus houi*]
CmedCSP16	KM365200	Unigene26507	144a	complete	19	1e-79	gi|723592548|gb|AIX97835.1chemosensory protein [*Cnaphalocrocis medinalis*]
CmedCSP17	KM365201	Cl5537	157aa	complete	18	7e-69	gi|723592548|gb|AIX97836.1chemosensory protein [*Cnaphalocrocis medinalis*]
CmedCSP21	KM365205	Unigene22979	128aa	complete	18	2e-77	gi|723592548|gb|AIX97840.1chemosensory protein [*Cnaphalocrocis medinalis*]
CmedCSP23	KP975086	Unigene34455	128aa	complete	18	5e-56	gi|524903053|gb|AGR39573.1| chemosensory protein 3 [*Agrotis ipsilon*]
CmedCSP24	KP975087	Unigene36321	127aa	complete	16	2e-71	gi|82792665|gb|ABB91378.1| chemosensory protein [*Helicoverpa assulta*]
CmedCSP25	KP975088	Unigene38001	127aa	complete	18	6e-59	gi|552955226|gb|AGY49267.1|putative chemosensory protein [*Sesamia inferens*]
CmedCSP26	KP975089	Unigene36705	124aa	3’ missing	18	3e-40	gi|122894082|gb|ABM67687.1| chemosensory protein CSP2 [*Plutella xylostella*]
CmedCSP27	KP975090	Unigene37675	124aa	3’ missing	16	5e-44	gi|122894086|gb|ABM67689.1| chemosensory protein CSP2 [*Spodoptera exigua*]
CmedCSP28	KP975091	CL5373.Contig1	124aa	complete	18	2e-54	gi|712120529|gb|AIW65099.1| chemosensory protein [*Helicoverpa armigera*]
CmedCSP29	KP975092	Unigene10982	123aa	complete	19	3e-44	gi|158962509|dbj|BAF91715.1| chemosensory protein [*Papilio xuthus*]
CmedCSP30	KP975093	Unigene38015	123aa	3’ missing	18	2e-53	gi|524903217|gb|AGR39578.1| chemosensory protein 8 [*Agrotis ipsilon*]
CmedCSP31	KP975094	Unigene37928	121aa	complete	16	7e-62	gi|365919036|gb|AEX07265.1| CSP2 [*Helicoverpa armigera*]
CmedCSP32	KP975095	cl3683.contig2_	120aa	3’ missing	16	1e-73	ref|NP_001140188.1| odorant-binding protein 4 [*Bombyx mori*]
CmedCSP33	KP975096	cl1009.contig2	120aa	complete	16	6e-51	gi|564969460|gb|AHC05672.1|chemosensory protein, partial [*Chilo suppressalis*]
CmedCSP34	KP975097	Unigene37060	112aa	3’ missing	16	4e-50	gi|365919040|gb|AEX07267.1| CSP6 [*Helicoverpa armigera*]
CmedCSP35	KP975098	Unigene36273	99aa	3’ missing	16	2e-29	gi|405117272|gb|AFR92092.1| chemosensory protein 8 [*Helicoverpa armigera*]

### Validation of tissue- and sex-specific expression of candidate chemosensory genes

**Fig 8:**
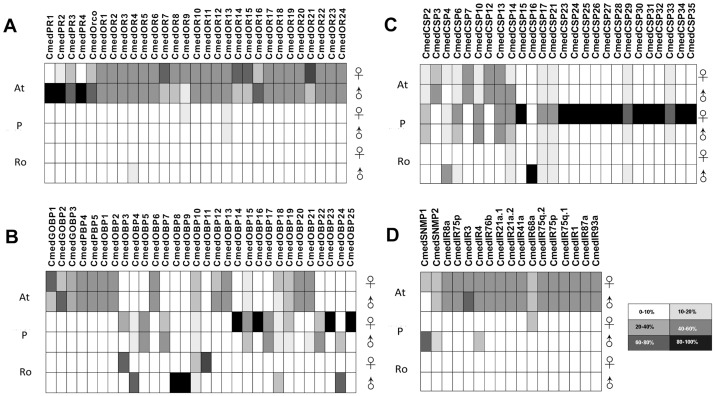
Comparison of OBP, OR, CSPs, SNMPs, and IRs expression based on FPKM values in male and female adult C. medinalis chemosensory tissues. (A) The common set of ORs expressed in each tissue in both males and females. (B) The common set of OBPs expressed in each tissue in both males and females. (C) The common set of CSPs expressed in each tissue in both males and females. (D) The common set of ORs expressed in each tissue in both males and females. The antennal libraries included both antennae, the protarsus libraries included the tarsus of six legs, and the reproductive organ included the last three abdominal sections.

In *C*. *medinalis*, all 29 ORs were expressed in antennae, 12 of which showed sex differentiation, with the expression level of CmedOR7, CmedOR14, CmedOR15 and CmedOR21 higher in female antennae than in male. Female-biased OR expression, as quantified using RNA-seq data, has also been reported for ORs expressed in the antennae of the adult mosquito, *Anopheles gambiae* [54] and other lepidopterans [55]. Three putative ORs were also expressed in legs and reproductive organs, respectively (Fig 8A), and CmedOrco was specifically expressed in adult antennae. Functional characterization of these male- and female-biased ORs, as well as members of the PR clade, remains to be conducted.

Unlike ORs, the expression of the IRs appeared to be similar between male and female (Fig 8D). We observed that some CmedIRs transcripts were not specific to chemosensory tissues, as 13 of the 15 candidate IRs showed exclusive expression in male and female antennae. Similar results were also observed in *S*. *littoralis* IRs [56]. The relatively high sequence conservation and expression of IRs implies a probable functional conservation. The antennal IRs are a novel group of chemosensory receptors. Additionally, CmedIR4 and CmedIR68a showed considerable expression in the male and female tarsus, respectively. In *D*. *melanogaster*, most of the 15 antennal IRs were found to be expressed only in antennae, two were also expressed in other tissues, such as the proboscis [29]. And CsupIR3 and CsupIR64a have considerable expression in leg [37]. Thus, the chemosensory function of ORs and IRs might not be restricted to antennae (Fig 8B). In Lepidoptera, legs and ovipositors are known to carry contact chemosensory sensilla. For example, the ovipositor of the moth *H*. *virescens* has OR-expressing sensilla [57]. Taken together, these observations suggest that, although classified as antennal IRs, some IRs might be involved in functions other than chemoreception [56].

**Fig 9:**
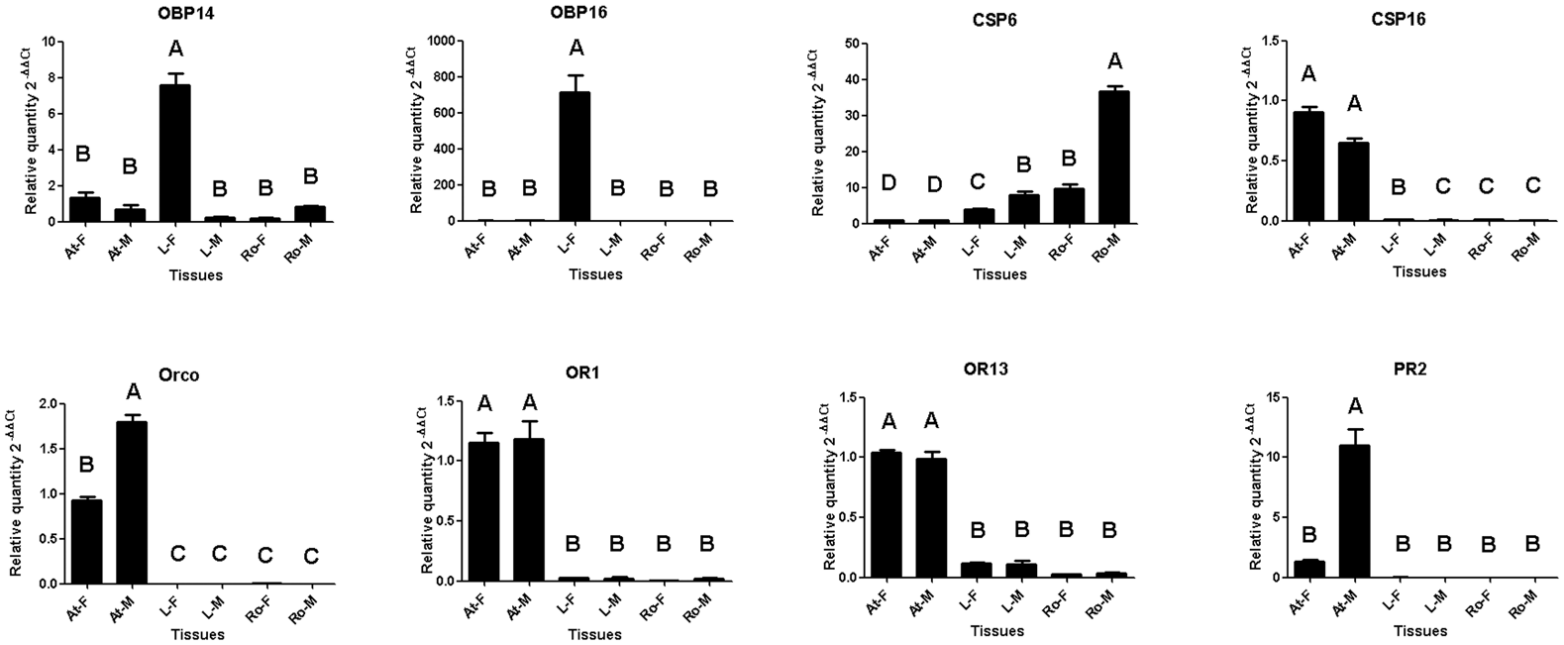
Expression levels of eight selected genes based on qPCR in different tissues. F: female; M: male; At: antennae; P: protarsus; Ro: reproductive organ. Different letters above bars indicate significant differences in expression levels between tissues.

Compared with the chemoreception genes of *C*. *medinalis* previously submitted to NCBI, most of those sequences were found in our data and the expression patterns of these genes in different tissues were nearly identical. In addition, we identified 13 new CSP genes, 11 of which were only expressed in the female protarsus, the remaining two were also expressed in the male protarsus and one was expressed in the male reproductive organs (Fig 9C). In total, 12 OBP genes were exclusively expressed in antennae and four in the female tarsus. In addition, CmedOBP6 and CmedOBP12 were also expressed in the male reproductive organs; and CmedOBP16 and CmedOBP24 showed sex differentiation (Fig 9B). The high expression levels in male antenna could help male moths to identify sex pheromones emitted by female moths. In many species, soluble PBPs in the sensillum lymph surrounding the dendrites are thought to transfer the usually hydrophobic pheromone molecules to the dendrite membrane of the sensory neurons [58], and are male biased in expression [59, 60]. Several studies supported the role of PBP in pheromone detection, since female moths release a blend of sex pheromones to attract males over long distances, and males detect the released pheromones with extreme sensitivity and selectivity [61], PBPs though are not the only protein that can bind sex pheromones, OBPs and CSPs also function in this. Labeling was observed in antennae sensilla chaetica, but not in olfactory sensilla or sensilla coeloconica, leading to the suggestion that in Orthoptera, CSPs are involved in contact chemoreception. This may also apply to Lepidotera as BmorCSP4 was higher expressed in contact organs (antennae, legs, and wings) than non-contact organs (head, thorax and abdomen); many insects have high expression levels in antennae, legs and wings but lower levels in the abdomen, thorax and head for both sexes [62]. Furthermore, among insects many adult females do not automatically oviposit once they have reached the spawning place, initial determination of host suitability may occur through the tarsal sensilla [63]. In *C*. *medinalis*, four OBPs and 12 CSPs were identified that were expressed exclusively in female protarsus, which may function in host plant discrimination. We speculate that prior to oviposition, females assess the suitability of leaf surfaces with their legs. This behavior may help to characterize the function of these proteins in future research. In the Mediterranean fruit fly, *Ceratitis capitata*, CcapOBP99d is abundant in the antennae, but also present in tarsi, wings and male abdomen [61].

To validate the expression patterns of candidate genes, eight genes including two OBPs, two CSPs, and four ORs were selected and analyzed by qRT-PCR. The qRT-PCR amplicons were sequenced directly after amplification and show ≧99% identical at the nucleic acid level with the corresponding sequences from the transcriptome, indicating that the assembly of the transcripts was reliable. The expression levels were consistent with the initial FPKM calculation (Fig 8), which further supported the validity of our data.

## Conclusion

The main objective of this study was to investigate the transcriptome of the major chemosensory organs in the rice leaf folder *C*. *medinalis*. Using RNA-sequencing, we annotated a total of 30 candidate OBPs, 14 CSPs, 36 ORs, 15 IRs, and 2 SNMPs in the antennae of *C*. *medinalis*. Most of the previously annotated *C*. *medinalis* chemosensory genes available in NCBI were also found in our dataset. Prior to this study, members of the major chemosensory genes had only been identified in the antennae of *C*. *medinalis*, and no ORs or IRs were identified. This strategy is particularly relevant for the identification of new insect chemosensory receptors in species for which no genomic data is available. The availability of our large antennal transcriptome represents a valuable resource for further studies on insect olfaction in this species as well as other leptidopterans.

Although the generation of gender-specific transcriptomes did not highlight strong differences between sexes, we found evidence for female-enriched ORs. A comparison of the transcriptomes in major chemosensory organs from males and females that have encountered diverse experiences might lead to the identification of more regulated genes, such as candidates for genes involved in gender-specific behaviors and make it possible for further research into the *C*. *medinalis* olfactory system at the molecular level. These studies can also provide information for comparative and functional genomic analyses of related species.

## Supporting Information

S1 Fig The FPKM of candidate chemoreception proteins in different tissues. (TIF)

S1 File Amino acid sequences of *C*. *medinalis* olfactory genes. (DOC)

S1 Table Primers used in the quantitative real-time PCR analysis. (DOCX)
