# Supplementary figures and images for: Identification and Comparative Expression Profiles of Chemoreception Genes Revealed from Major Chemoreception Organs of the Rice Leaf Folder, Cnaphalocrocis medinalis (Lepidoptera: Pyralidae)
Source: PLoS One. 2015 Dec 11;10(12):e0144267. doi: 10.1371/journal.pone.0144267 (PMC4676629; doi:10.1371/journal.pone.0144267)

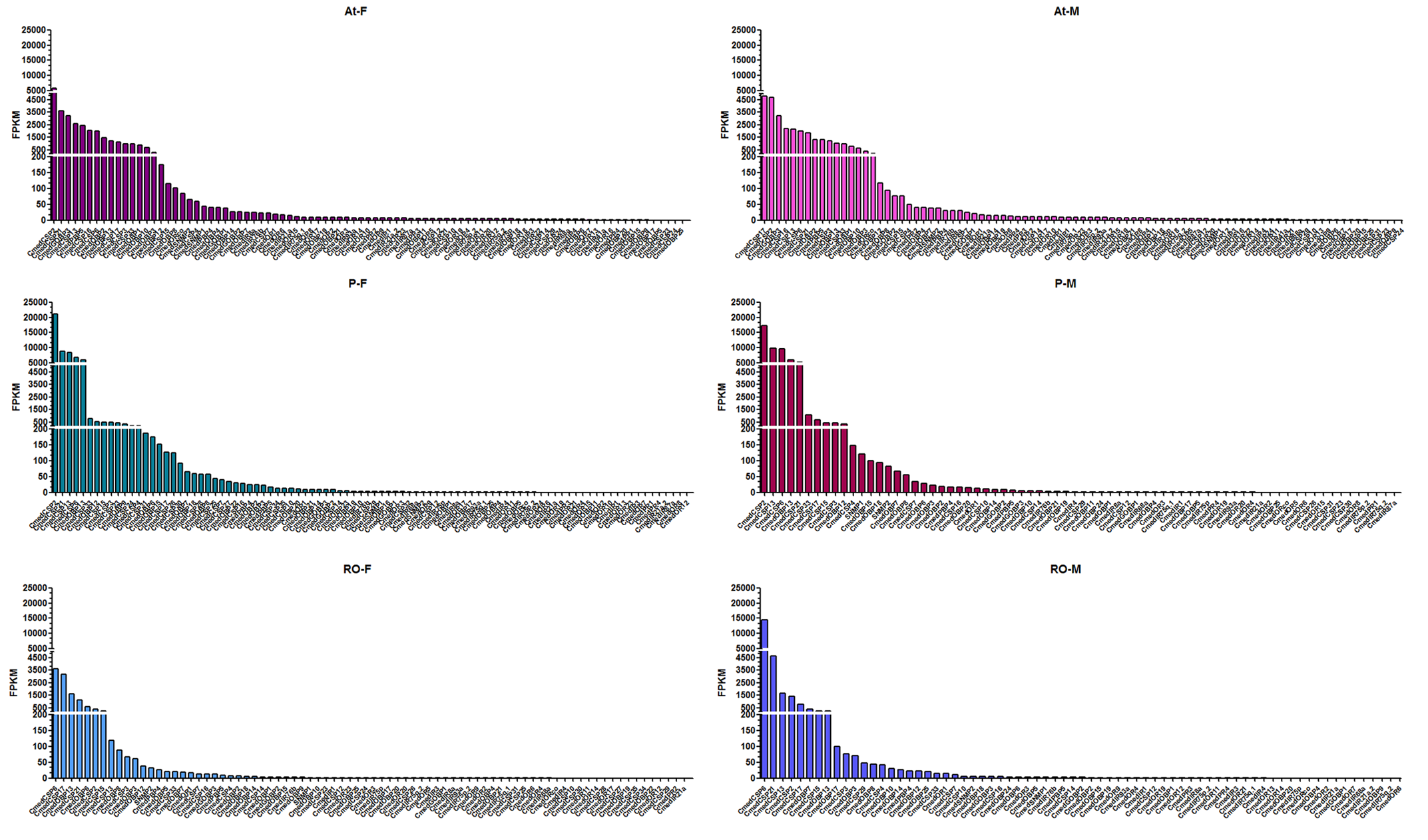

Supplement: S1 Fig — (TIF) [file pone.0144267.s001.tif]
