# Supplementary material for: Identification and Comparative Expression Profiles of Chemoreception Genes Revealed from Major Chemoreception Organs of the Rice Leaf Folder, Cnaphalocrocis medinalis (Lepidoptera: Pyralidae)
Source: PLoS One. 2015 Dec 11;10(12):e0144267. doi: 10.1371/journal.pone.0144267 (PMC4676629; doi:10.1371/journal.pone.0144267)
Supplement: S1 File — (DOC) [file pone.0144267.s002.doc]

**Supporting Information**

**S3 File. Amino acid sequences of *C. medinalis* olfactory genes.**

>CmedPR1

MLLHLFGHFMFIYCRFHELDFTMLGDVYLTMIFTCLIIFRFSLLITEGYGKLFWSYLREFHLSHFKHRGEYVQQLCEKIDRLSYLFTLYQIALAMAGTVTFNITAFVINLSRGAFRTPRPENITLEFSVHFMWPGFTIEDHFYFTSIDNLFNSMLFGVSLCMQDLFLCLMIFQLIGHIKVLVKTLRSFPKPQRGAPLEYRRRNGSIYMVDIVKNFNAQENETIRKLIKECVDHHVMIVSFSDNISSFFGPMLGVNYLYQVICLCIMLMQCMMGPAAMLRYLTLTLGTLGQLLQFSIIFEIVGVESEKLKDEVYFLPWESMSVSNQKAILLFLRRVQTPIHVMAMGMTPVGVKTMGNIIKTTFSYYAFLRTSQGLK

>CmedPR2

MTAINKSGQRTEDPMTLKYMKLIRSMLITVGLWPGEAVAGKPHRIPFANMFITWQSCFCIYGELLFIYRRFRILSFFVLGDVCIAFSLTMLNLVRAVFPYSDTYGAIFHDFVSVFHLKHFKHKSEYARKTCETVDRISYYFSLYMTVIMVIGVSSFNLTPQYHNYQNGIFKDDAPENITIEFAVYYSFPYFEQEDHIAVSNLYNVFLSYICAVEVCILDLFLCVAVFQTIGHIHTLVNTLRSFPGHRKLQHPVQFRKSVGADSGSISVEIIRDFDDEENKIIKKLIKECVEHHFFIVSFTERLSKFFGPLLGFNYMYQTFCLCILLLQCMGGGGALMRYAPLTLITFGQLLQFSVTFEIVGAESEKLKDEVYYLPWESMSVSNQKAILLFLRRVQTPIHVMAMGMTPVGVKTMGNIIKTTFSYYAFLRTSQGLK

>CmedPR3

RYGDVMTNFLTNFHLIHFKHKSEYSKKIYEEVNKISLYFTRIMFGMTWTGVMSFNLTPLFLNYRSGLYHELIRGQATNLTMQFAVRYSFPGFEQEDHFLLSSLLNLLFSYMCGFTVCTVDLLLFIIVFQIIGHIRTLRHNLEVFPKPREMRDSLLKGIASDRVKFVRNFDDRENARIKTLLDDCVRHHLMIVSFTDEISSFFGPILGFNYLYHLVTCSLLLVECMEGKGAYMRYGPLTLSTLAQLTQMSVIFEIVGSESDKLKDAVYFVPWESMSVRNQKQVCFFLSRVQ

>CmedPR4

GVSFCMQDLFLCLMIFQLIGHIKVLVKTLRSFPKPQRGAPLEYRRRNGSIYMVDIVKNFNAQENETIRKLIKECVDHHVMIVSFSDNISSFFGPMLGVNYLYQVICLCIMLMQCMMGPAAMLRYLTLTLGTLGQLLQFSIIFEIVGVEVTIFLLLYY

>CmedORco

MMTKVKAQGLVSDLMPNIKLMQAAGHFLFNYHSDNAGMSTLLRKIYASAHAVLIVIHYLCMAVNMAQYSEEVNELTANTITVLFFAHSVIKLLFFAINSKSFYRTLAVWNQSNSHPLFTESDARYQQLALTKMRRLLYFICGVTVLAVVSWITLTFFGESVRLIANKETNETLTEPAPRLPLKAWYPFDAMSGTMYVVAFVYQIYWLLFSMAMANLLDVMFCSWLIFACEQLQHLKAIMKPLMELSASLDTYRPNTAELFRASSTDKSEKVPDPVDMDIRGIYSTQQDFGMTLRGAGGRLQNFGTNGSNPNGLTQKQEMLARSAIKYWVERHKHVVRLVASIGDTYGTALLFHMLVSTITLTLLAYQATKINGINVYAFSTIGYLSYTLGQVFHFCIFGNRLIEESSSVMEAAYSCQWYDGSEEAKTFVQIVCQQCQKALSISGAKFFTVSLDLFASVLGAVVTYFMVLVQLK

> CmedOR1

MTSEGRPRRYFALHYSLLRFLGLGWWHHPDEGDTRNFPGWYLYYSIITQLVWVVGFVGLETIDPFIGEKDIDRFMFSLSFVITHDLTLIKLCIFYFRNDQIQDIVRTLEIELYDYYQNDKKNQATVRITKILTSSFLFFGWITIGNTNVYGTIQDLRWKAEVAGLNDTSLKPPRTLPQPIWIPWKYQSDRAYISTFVLETVGLLWTGHIVMTIDTFIGSVILHMSSQFAILREALVTAYDRTISKLHDARQDIEEPIETSDGTTYDNEDGVHNAMEEIVFARYSAKEIDLALEETLKNCFRQHQVLIRCVEKFAATYSYGFMTQLLSSMAAICVVMVQVSQDASSFKSIRLVTSLAFFMAMIIQLAIQCFTANELTLQAGRVADAVMQCKWERMSPRLRRQLVLVTVRAQRPLRLSAAGFANMNNDCFLAIMKAAYSYYAVLSQKQNI

>CmedOR2

MDFFEIEDKENAEKEDKEYKFKPFHLTFKWIASFLMLGLLYPNPRTDKTRFMLIMALLFVTLPVIVVINIDIFVCWLKRDMFNIIRHSTIIGPVMGAFFKMFVLFHKRVQAKLLIDEMTEDYESYNSLPPTYQEMAVRTIRTCKIYTLWWGLLITTTILMFPVLALGSTLYSHLFESIPRRYMLHDVNMPFMPTEARFESPYYESIFACMCVCAVISVLNYTSYDGLFCLITNHACLKMSMYCLRLEDAFKQDDPDVMWSELVTVIREQMKTFRFSALIQNTFNTWLGTILISTMIEIGSLLFHISAGYGFDVRYAVFSITSVVHIFLPCLYAANLSAMSTDTATLVYCSGWERACPPRIRRLLPFVVARAQSPIEINAFYMFRYDMMLFVNIMRTSYSMFTLLNTS

>CmedOR3

MDSRLFKAIKAFLMKENFDFTRPDMDLYIFHPQLRLFIAPMGIFFNNTNSLLRFVWPFLSVSLSITAIVLEMIFVYHGLMVKDYAFATECFCYFIMLGIIPLVYGCIIFNRSSVLELLEDMNKDFKLICKLDARYRDHFMKGQLLIWQLCFIWIWFTFVIVVMYCIMTMGPLLYLSLFATQDEHKVRPLMFPMWLPKDDPYRTPNYEIFLILQVNFCIMYIQTFAVYVYI

>CmedOR4

YKDAVRNIVDGYLACDARNTQGSRFSQNQLKTLRAVKKRALIFWFVIIGNGVIYITKPILTPGRHLMEDCFIIFGLEPTFESPNYEIAFLLTCCGVFTTCYLPANITAFLIVVIGYTEATMLALCEELLHLWDDAHEAYNNHKQLSITSRDHYAGNEYNSRTIFVNKYVKQRLDEIAKIHMTNINLIHQIEVVFRGAIALEFVLLIHGLIAELLGG

>CmedOR5

RFLVECHWWFIVANMVLVDGLASTSLVYVGYKFRLVCIHFKRLRERTLANVKVKPERECMEEFEKDFVAGIRLHEEALWCARNVQSTMGKTYGVQIIESTTLLVICLIKLVTFERNMTFLVANGFYIACLLTLNGAYMMAAGDVTYEASLLPTSIFHSGWELVRTRRQFYALAVVAIMNSQKPVYMTAFGVITLSHRNFISVMRSSYSFFAVMY

>CmedOR6

MEWKNWLEAYPEEYQKPLIETLDLLRKGNIRLLGPNVPFVKKYWHFFYMIPLVTVHYASMITHIVLTEKFDHFQRADLPMFLCGSACIIKTIIIYTKQEEIREFIIHLGSSWRTDDLNDAQLKLKKDAMRHLSYAVIAFCRLGIIFSVQFIMWPLCDTVIRRLLLNQDIELQLPYSCVYPFEIVDWPVYLAIYALQVFCTLYSTSYIYIGT

>CmedOR7

VYVYIQFHVLLHNYYLLELVILSFGVIFDGLDVGVAYLPVRDERRIAVQRTLNKRMERIVTWHNSVFKSIAKISKIQGAPLVYQVMFSSAAVCLMMYQIADKLDNGVVDILFIMLFSAATIQLWVPCHLGTMLRNKAFEVADACWHCGWHETLLGRLLKTDIMIVMVRAQQPLSIKFTGLPNLSLETFSSKMSSAYSLFNMLRQYN

>CmedOR8

WVPWFTVYIHSVAAAYITIIYSLIMDGLFFVLVTNICINFSILSNDILKLKENNFRNINDCIRTHQYLIKLSEELNDIFEAPNFFNVLFGALQICALGFCITAGDWSLVPAYLLFLSTVIVKLWTTSYFGEKLVEKSTEVNEAILNVDWYEADLKIQKTILLIIARSQQPQRLTAYMFSTVCISSFSKIMSNSWSYFSILRSAYQKPEG

>CmedOR9

EMLHIWSDANKFYQDNCKDDSSVVVVNKRRNENETAYQSHKRIVNSFIKQRLYELIKIHTTNIGLVQKVEMLYRELIAAEFILLTFGITVELLGRLENTYLQIPFALMQVAMDCFSGQRVMDASLTFENAVYACKWENFDVANMKTVLLMLSMSQKTLKLSAGGVTMLSFTSLAGVIRIIYSGYTALRQLY

>CmedOR10

HYDREMSHRFTEKKSHFDIYSPEFDAATYDALRDCARVCDVVNRYKNKFESFVSPLLALRVVQVTLYLCMLLYAATLKFDMVTVEYLAAVALDTYVYCFYGNQIILQADRVSTAAYQSAWHTMGARPRRLLLNILLANKRPVAVRAGSFLPMDLHTFVVIIKTSFSYYTLLVNVNEQK

>CmedOR11

FRLLSHRIQNVVSSPEQTRRGLKAAVRRHQELIDLVDQVELLYSKSTLFNIVTSSVLICLSGFIITVLDDISVVVTFATFLFMNLSQISLLCFFGDMLMRSSTQISSAVYHSLWYETDPRTKKSMLLILMRAQKPCKLTACNFADLNLTAFTTILSRSWSYFALLKTMYK

>CmedOR12

RTISKETLNETYEAILSKLLLECIVHFQKIAETMALLQSVFGLAVLMQFTVGGWILCMAAYKIVSVDILSFEFASTTLFIVCILTELFLYCYYGNEVTVESDRVVESIYAMEWLHAPLRFKKSLVLMMERAKRPLRPAAGRVIPLSLNTFVTILKSSYTFYAVLRQTK

>CmedOR13

ISKETLNETYEAILSKLLLECIVHFQKIAETMALLQSVFGLAVLMQFTVGGWILCMAAYKIVSVDILSFEFASTTLFIVCILTELFLYCYYGNEVTVESDRVVESIYAMEWLHAPLRFKKSLVLMMERAKRPLRPAAGSIIPLSLDTFVTIMKSSYSFYAVLRQTK

>CmedOR14

YNLTAEQEKVVQERITKCVAEHQRALQSALLIQTCFSEQIFAQFNVSLVIICVTAFQLVSQTGNLVRLMSMGTYLLNMCFQVFLYCYEGNQLSEESSEIAGAAYEAPWYVMSPALRRSLLIIMTRSRRIARITAGSFTTLSLGSFMAIIKASYSLFTLLQQVEG

>CmedOR15

VVLTLYLNIQFLLLQEDLIKINLKDNKKRLEHSSAEDFDESAILEQKPKLRIEDFVRLHQDAILLSNKMNAACGQLNFTIMLFVTLIISLLAVSIKETSGIGNLVNCCGVTSVVMFNIYFLCYCSERLCSSSSGIAIAAAQTLWYNGDLRYQKIIKFIIVRAQ

>CmedOR16

KSNVHLLKGLVKDHQYVLKLSQDLEEIFKLPNLYNVLVGSLEICALGFNLTIGDWPQVPGSLLFLLTVLLQILMMSVFGEKLITESRKVGDCAFYSQWYDMDQKSKKTILFIITRSQKQQQLTAYKFSVISYGSFTKIISTSWSYFTILRTVYTPPESLQVE

>CmedOR17

ENLKLEDLKDTSKEEFLKRQLNKCLIHYDLILKYCEHIQKVLDFSLFAQFGTAAATICISMCTFLRPMTNAYKIFIWFYIGSMMMEIFLPSLLGAELTAESLKLVPSAYSSDWIPRSESFKRSLRLLVERANRPIVITGLKMFPLSANTFIAIIKTAYSFFT

>CmedOR18

ALDRLRSCIIRHQSVLQSADSIQELFSASILAQVTVSMVIICVTAYQLAFETKDLVRFISMLAYFLCMTLQLFLYCYQGNQLSEESTQVAGAAYETPWWYCPAEFKRSLLMMMTRSRRMARVSAAGFTTLSLSAFMSIMKASYTFFTVLR

>CmedOR19

NIKLHQRLIRYCNDLEVAFSLSNLINVVLSSVNICCVVFVIVLLEPFLAISNKLFLGSALIQIGMLCWYADQILHANADVASAAYNSRWYRTSARCRRALLFLIQRAQKPIAFTAMGFTDISLVTYSSILSKSYSYFAL

>CmedOR20

VLCLVGIQFLSIENPSSHPMQIMWMAIYLTCMLIEVFILCWFGDELIWKSIALRQAAFDGPWLGTDQKTTIFIVIFLERCKRPLRVTAGKIFTLSLDTYTILINWAYKAFAVISNMKK

>CmedOR21

GGWILCMAAYKLISLNVLSIEFASMTLFITCILTELLLYCYYGNEVFEESDRVVQSVYGMEWLHAPLRFKKSLVLMMERAKRPLRPAAGSIIPLSLDTFVTIMKSSYSFYAVLRQTK

>CmedOR22

ILCTSAYRIVNANPASIEFVSMIMYVMCIVIELALYCYYGNEITYESEKLMESAYAIDWLQMPINQRRSLIIFMERIKKPILPMAGSFIPLSNSTFISIIRSSFSFYAFLKNSEH

>CmedOR23

MWFHLLRNAKKSKESTLDSEPIKNYIHFLEIPLKIVGCWDWYKNPTKDREIIINNLYLGAVLFSIINVPITLFVHVYIEWVDIMTSLDILADCLPFAVS

>CmedOR24

CALRIIAEDTQGMQIVSIIAYLLVMLSQLFICCWCGHELTASSEDLHSVMYKCVWYEQDVRFKRDLCFAMMRMSRPLVLRAGHY

> CmediGluR1

MATGIELILSTVCNATFCEAIYDNFPLDAARSRKAELYDALASEFNGKHLKIGTYNDRPMSWVEQGANGTLIGHGVAFQIVEILKKKLNFTYEVVVPEQNFQMGGSKPEDSLVGLANNSLVDMVAAFLPNDVKFRNKVAFSCGLDRGVWVMMLKRPKESAAGSGLLAPFNNAVWYLILVAVLSYGPCISLLTRLRTRLVPDGERYISLSPSFWFVYGAFIKQGTNLAPEANTTRVLFTTWWIFIILLSAFYTANLTAFLTLSRFTLDIETPQDLYKKNYRWVSAEGSAVQNIVTNPNEDLYYLSRMIATGRAEFRSMSNDSDYLPLVNGGAVLVKEQTGIEDLMFTDYLQKAREGVAEAARCTYVVAPNYFMTQLRGFVYPRNSKLRDLLDSPLMYILQGGIIDFLKTKDLPSTKICPLDLQSKDRQLRNSDLMMTYMIMVTGLAAAIAIFIGELFIKRYILKLTKGKGKTKRKKTKFEKHPRVYNYDDSRPPPYDSIFGHNPKIKTDTAKSKIINGREYLVIEAANGDTRLIPVRTPSAFLYHLDRHVKK

> CmedIR1

VKVSDSVVLLDRENEEESYRKLCSALSQGVSLIVDLSWSPWAAAEDTGIPLVRVSLGSQQMMAAGEQYMASRNATDAAVIAESEADVDRTLYELLGRSNVRLWVHAGLTRDSARALKTMRPEPSFYVLVGESGFVMDTYRRAVKEKLVRRDYRWNLFLTDYSGDSLDVTQLVLPAMVLHVDPTECCKLLGLKEECNCPSDFKRIPHIINALVLYIVDTYIAIDSESVTTKVDCDDIQVEDLAAVKDKLTRQFVEDARMNNESLFYWDEARSGLFLRSTFVLSTYKPDSGLETVASWSADEDYKLLPGVTLDPLRLFFRIGTSPAVPWTLPKLDPETGEQMLNEDGQPLYEGYCIDLIAKIAETMEFDYEIITPKTGSFGKKLPNGSWDGVVGDLMRGETDMAVAALTMTAEREEVIDFVAPYFEQTGILIAIRKPIRKTSLFKFMTVLRTEVWLSIVAALVLTGFMIWLLDKYSPYSARNNPDAYPYPCREFTLKESFWFALTSFTPQGGGEAPKALSGRTLVAAYWLFVVLMLATFTANLAAFLTVERMQTPVSSLEQLARQSRINYTVVEGSTIHQYFINMKFAEDTLYRVWKEITLNATSDQSQYRVWDYPIREQYGHILLAINASGPVPDAKTGFEQVNEHTDADFAFIHDSAEIKYEVTRNCNLTEVGEVFAEQPYALAVQQGSRLQELLSRALLDLQKERFLEQLAAQYWNESARQACPDADESEGITLESLGGVFIATLFGLGLAMITLAWEVFYYKRKEKNKVQALDVKLEKAAFVEPKNTEKAVRFRKKGKGKKGELSKVGKLDEEKVKKGVTIGDSFKPASEKMGVSYISVYPKGEFRP

> CmedIR2

VDPSSDDQRGITALFKCPLLPDSDLVLAERVENGFIMTELHRTSANSSMIFTSRGHYNGKLTDVRPHRELYRRRRDLRGHTLTMSNVIQDSNSTLYHLPMEDRLEPQFDSISKICWMCTTLLFKMLNATPHYIFNHRYGFLNNGTWLGMVGDVASGLADLGTNLLLNHDRIQVVTYTDMMAPFELRFVFRQPPLSHSSNIFSLPFSSGVWLSIVMCSAVAAATLYLSTKWETRLEKSPSQLDGSIGDALLLTMSAVGQQGCYQEPTKLSGRIITWVFFAALMLLYAAYSANIVVLLQAPSNSIRTLAQLASSGITLAAHDVDYN

> CmedIR3

LAIVVCSAMAVATLFLSTKWETRLETSPTQLDGSIGDAFLLTLSAVGQQGCYQEPRKLSGRIITWVFFAALMLLYAAYSANIVVLLQAPSNSIRTLAQLASSGITLAAHDVDYN

> CmedIR4

LNDAMHPIRSLETNEDEVKALMNLKKLYAAFIALAIGYSLSSLCLIGELIHWHLVVKRDPEFDKYALDIYYMNKNKKQ

> CmedIR21a1

TRLLIGWYWIFTIIITSCYTGSIIAFVTLPVFPETVDTIQQLLAGFYRVGTLDRGGWERWFFNSSDQKTNKLLKKLELVPNVEAGIRNTTKAFFWPYAFLGSKSELEYIVQANFSVATSKRAMLHISDECFVPFGVSMAFPSRSLYSSKLSGDLRRMFQSGLIEKIEDEVRWEMQRSSSGKLLSVGSGSLKLVSAEEKGLTLEDTQGMFLLLAAGFLLAATALISEWMGGITRRCRLGRKKPPSSANSKEQLIIPPDLEREIDGTESRLNFDSRSTTAGSRDTLDGQVINLTEENITVHEQLDAGRWDSRRSSSVDLDKEVQEIFEKDLRRRKIIKEGSIEVQEIENREPTASNGAFGDRLT

> CmedIR21a2

KSKESYLFQNLLKGFPVSYVHGYIGDDNKLKEPELLVPGKQCLHFIIFSSEVKSSVRILGKQSESKVVVVARSSQWAVQEFLSSSTSRMFINLLVIAQSFKDDNDETMEAPYILYTHKLYTDGLGASQPVVLSSWTHGKYSRDVNLFPPKMTDGYAGHRFIVAAANQPPYVFRRIQSDRDGGNPRVVWDGIELRLLGLLAERNNFSIEILEPQEPNLGPGDAVSKEVTSGRADIGIAGMYFTSERTQGLDMSFSHSQDCAVFITLMSTALPRYRAILGPFHWHVWVALTFTYLIGIFPLAFSDKHTLRHLLNDSGEI

> CmedIR41a

MLISQSQFDVGTSWRVRSITGWMLLAGLILDNAYGGGLASVFTVPKYEKSIDTVQDIVDRRLDWGATHDAWLFSLLLTTQ

> CmedIR68a

IRLLTGWYWLYCILVVVSYRASMTAILANPAPRVTIDTLKELLESKITCGGWGTETKKFFQESSDEIEKIGQRFEVINDPFEAANKVAKGIYAYYDNQDFLKYISVKRKNIDMNIESNVNATLNASEIFSVDVERNLHIMSDCVVNTPI

> CmedIR75

YSYMNASRTLIFTETWGYYRNGTHGGMIAEMTKGDADIGGTVLYVTKDRLEVVDYLSSPIPISIKFVFREPPLSYQNNLFLLPFETAVWYCMGAFVLVLGFILYITALWESKMMGPSSINPTDPTALKPSVSDMAFLVISAMSQQGSSTELKGTLGRIVRFILFLAFLLLYSSYTASIVTLLQSTSDQIRTLSDLLNSRLELGVEDAPYNRYFFQIATDPVRKAIYQTKIAPRGTEPKFMSLEAGVKKLQTEAFAFNMNKGIGYKLVERYFHEHQKCGLQEIEYLYTMTTYITCRKESPYKEIFKIGLFRIRENGLSDRENRLIYARKPACRARGGNFVSVNMIDFHPVVLMYLYGILLAFAFLAIEILVHKKQLLQCRVRNKAAGSLEPSVEKAMTALV

> CmedIR75p

PILVSSELVLAERHKEHITMTELHKPSVNHSLQFTPRGFYNGSLFDTRPHRELYRRRKDLMGHTLIMSNVIQDNNVSKEHILNEDRLDLHNDAVAKACWVAARHCFEMLNASENHIFSYRWGYKVNGEWSGMINDIKTKKADLATNCITYKDRLEVVSFTDMVAPVHMRFIFRQPPLTYTANIFSLPFSTDVWVAMAICSAAATVMLFVTSMWEVKMERNPTQLDGSFSDALLLTMSAVAQQGCYIEPSRAPGRIVEWSLFTALMALYAAYSANIVVLLQAPSNSIRTLSQLASSKITLSANDVDYSRFVLNKYNDSLHVGIYKRVTPDGGTPNYYQIDKGVEKIRQGLFAFHSIVEPVYRRIEETFLETEKCDLSEVDFVNGFDGFIPVKKDSPYLEILRIPFKRIRESGIQNALTRRFLVSKPRCVNSMSTFSSVGLLDMRPVIIFMLYGIIFSVMIFLAEVV

> CmedIR75q2.a

KSDSGVYVVADIIRAMEKPSTVVATLCWVTDEKLRFYYAVTDRERFSRITTAQLDGINHMKEDYGQDQHIVFVADLSCPNISVFFDKKRTENYFRSPFRWILIGGLMEDDDEIVPKAIAHVDVLPDSHVIVVRQVKEDIYDIHSIYRISADTDWRTELYGTWNKENRFVVTNPYMESIALQRLDLLGFEISICYVLTDNDSINHLTDEVNDHIDTITKVNFPTTNHLLDFLNASRKYIFADTWGYRVNGTWNGMTGYLIRQEVEIGGSPMFFTSERISVVDYIASPTPTRSKFVFQQPKLSYENNLFLLSFRTSVWYSSTGLLFLLLLALFVVAVWEWKKNAKDTQRAHDSGTLRANLVDVTILIFGAICQQGSPVELKGSLGRVVMLILFLALMFLYTSYAANIVALLQSSSSQIRNLDDLLHSRLKFGVHDT

> CmedIR75q2.b

SQIRNLDDLLHSRLKFGVHDTVFNKYYFSTATEPVRKAIYEKKVAPPGTVPRFMSMEEGVKKMRKGLFAFHMETGVGYKFVGKYFNEGEKCGLQEIQYLQVIDPWLAVRKHTPYKEMFKIGMKRIQEHGLQSRENWLLYEKRPKCSGRESNFVSVSMVDCYPALLILTYGTILSLMLLACEFLYLKRQ

> CmedIR87a

MFPLDLKGCPLVTYAVVSEPYVLPPVSQVENSIYNDSYEFQKGGEINLVRIISQFTNMSLIMRTSDIPENWGNVYWNGTASGAYDVLRSDSVDLVIGNIEVTRTIRRWFHPTVSYTQDEMTWCVPKAGQASTWNNLVIIFQWSTWVATFGSLVVMGLLFHYLYYKENNKQVTKWPTNSLLMTFSMLLGWGASFEPKSATFRILIFGWLCFSVNMGISYESFLRSFLMHPRFEKQISTETDLIQSRIPLGGREIYRSYFETNNASSFYLYRKYNSTTFAEGIKRAAKDRNFAVVSSRRQAVYQDQKLGRGKHLIYCFPESDNLYKYGVVLLARKWFPMLERFNNIIRSVSENGLIDKWNEELFIHTASAEGTNEIEPLSIQHLLGAFMFIGFMYAASVLVFVLELFFGEIQKRKGKNFWQIVFRKKITFSR

> CmedIR93a

MQTWLVVAMCVVGVSGEEFPSLITANASIAVVLDRQFLGEKYQAVLDELKDYIKELARVELKHGGVVVHYFSWTTISLKKDFLAVFSIASCEDTWSLFTRTEEEELLLFALTEEDCPRLPTDSAITVTYVEPGEELPQILLDMRTQRAFKWKSAIILHDDTLSRDMVSRVVQSLTSQIDEGATTTTVSVSVYKMKHEINEYLRRKEIQRVLSKLPVKYIGENFMAIVTTEVMTTMSEIARDLLMSHTLAQWLYVISDTNIHNGNLSSLVSALYEGENVAFMYNITDDDPDCKNGLMCYCQEMMNAFISALDSAVQDEFDVAAQVSDEEWEAIRPNKIQRRSMLLKHMQQHIATKSSCGNCSTWRALAADTWGATYRSFTETDMMAKDPGNVTTVGVIEHVTMLHVGFWRPIDAIRFEDVLFPHVEHGFRGKELPVITYHNPPWSIIKTNESGAVVSYTGLVFDIVNQLAATKNFSIRVILPSNVKYLKPNETVVGMTHNHDALVTLAAIAKGQAAIAAAAFTVLADPPPGINYTLGVSSQPYCFMIARPRELSRALLFLLPFTTDTWVCLGLGVILMGPTLFVIHRLSPYYEAMEMTRQGGLSTIHNCLWYVYGALLQQGGMYLPRADSGRLVVGTWWLVVLVVVTTYSGNLVAFLTFPKQEIPVTTISELLENRLTYTWSITKGSYLEMELKNSDEPKYTSLLKGAELRSEAGSVDSNQSPLKKQLKRTREQRHVIFDWKLRLSYLMRAEHMLTDTCDFALSVDEFMEEQLAMVVPAGSPYLPVINKEINRMHKAGLITKWLYAYLPKRDRCWKTSSVSQEVNNHTVNLQDMQGSFFVLFLGFFSASFVLLLEWLCNRRKLRSE

> CmedOBP3

MLVKSVLILSTVLLSSDAAFVDTLKKCGKNDGECHREMIQNVLKEASKPGIQELNIPPFDPVVLKDVSIPILNVVDLELADGVVRGMRNCVVNSFVTAMEKGKASMDITCDLVVKGKYKAFSNSPLVKSVLGADKLRGDGNAKLKIEQLNVKFDFHFFIEERDGETYIKCKDKKLDYKYEVKGKVVFAADHLYLDDKDASEVIVNLLNQNWRLVVESAGQQLMGRAMDRIKVFFTSFFGSVPTKYYIIDDLTPYVK

> CmedOBP4

MNIFIKNTIVLAILSTCCYGLVDIEKYLKVCDRNSAEVNDCLTEAVQEGLAALADGIEEIGVPPIDPYRQKDLRIEYKNNQILAKLVAKDIYVEGLKTATVHDARLRADDDRFHLELDLTTPRVNIRGQYLGEGRYNSLQIKAHGHFSTNMTNLVYTWKLDGVPEKNENGTFVRITDFYMRPDVGSMKSHLTNDNPDSRDLTELGNRFTNQNWRLLYRELLPYAQANWNKIGISVANKIFLKITYDQLFPSKS

> CmedOBP5

MIQYSLWVVCVFCILVNADPVSFINKCKADDDKCIKETTRVVIPKFTDGLPEYNVETLDPVFFKNIDSSSPTLKLLLDNVTVKNLKKCIPKSVRRDAQKAKLYLKIQCDATLDGHYDMNGRLLILSISGNGKIHVDLRKAVFDIEIDLMDKKGKDGKNHWQVKSWKYTYELNDKSTVYFENLLSGNPALAEAAKNVIAESGNEIIREVGSPVLKAVIGRIIENILHFFRAVPIEDLTLDN

> CmedOBP6

MGSPSFSAMWAVVLLALLPALVKCSGEGNIRLLEEEVANAMNACVQPADKKEAAPPQRQRRSEDYPRIDNNDSSSQNMYSHERRNTSDLQDTYVLNGSDYDWGYGDNRQARDRNSSEVYGNRTKRSEPLLNKPDSDQCLSQCIFANLQVVDSRGIPREAELWNKVQSSVTSQQSRAALRDQIRACFQELQSEAEDNGCSYSNKLERCLMLRFSDRKNDSGAAQTQSGGQKT

> CmedOBP7

TLLTTLFLAPVTKCDAKDSKCLKKSSQALIPSFAAGIPEYGVQTLDPLHIKTVDASSPNLNLEIKDMVVTGLKDCVAKKIERNETNLFTRILCTTKAEGQYTMHGQLLILPIEGNGRAEVKLNKIQMEVQHNMITKTGKDGKKHWQVKSYSYDFQLKDRAYVHFENLFSGNQLLAQAAQEIISSNGNEIVMEVGPPVIKAIVDKLVDSVNNFFKAVPLEDLEL

> CmedOBP8

MFALTLVFLATLSSLQGEAIDKEKDRPRKGPVLSEKNKMIGIDAVHDIKIDKDTIITRNLKLDKKKSAKAAAGHHAADEARAPDWSYDSIPQGLSGYVADFKRNMTECLKETQAQDKRPVKRISPKQESPVHGECLIACVLKRNGVIENGKINKGNLISLVGKFYAKDTKLMKKLEKNLDRCVEMSAAYRGDECALAARLNACTNDLMISNKHKIQVNY

> CmedOBP9

RPREPSHEMNRAFTNFVTIILFLLFVSAFYLVISFQPMSKEEHIEKINKMNEEVEPFRKNLTECARQVKASMADVENFLKRIPQSSMQGKCFVACILKRNAIIKKNKVDQHTLLQLNRAVYGDDSEVLSRLKTAIGECDQVVEGIFEICEYASVFNDCMHMKMEHLLDKVTMERRMEALGQMTADPDEWTDDEEEMLKLVKDEL

> CmedOBP10

MCKVSVLFLFVCAVYVEVSAKPPEPPQVLCGEIPSKIFSCLNVPIIGKDASDCQGNNSCEKLTCYFRQSGWMTGPSVNKAKLREVFDMYGVSHPEWAPLIEPAKAACATYDLPAQGYYLNCPAYDVVTCWLHYLIKNAQPSQWSTSPKCEAVRRFAASCPLCPSECFSQQIPIGSCNACYALPRSP

> CmedOBP11

MFRFILAAILALTVADEQTVLLRVIDAELLQDTSRSRGATLKPISACCDIPELGDPKPLSECSNPKLSGPCNDIQCVFEKSGFLVDKNTLNKDVYRRHLRKWADSHKDWSKGIEKAITDCVDKDLRQYLDYPCRAYDVFTCTGIALLKKCPDHAWKCGTISITPIVAPKP

> CmedOBP12

QRTCDSTVVAGVCAAAIAHHQVICLLVSLLSKARTPLEIRQFMMSKSIECTKDHPVSTEEMTTMMDHHLPNTKASNCFIACIFKKFGWMTEKGMYDLNAANLFAEDEYKDDATQIENAKKVFDACKGVNDESPADGEAGCDRANSLAKCLLEHGPKMG

> CmedOBP13

MYRAVTLCGLFFMALTPYFANAMSEEQKARIREHFETIGMQCITDNPITEQDINDLRGKKPPSGAGAPCFLACVMRNIGVMDDAGMLQKESVLELAKKVFEDEEELKIIADYLHSCSSVNSASVGDGAKGCERAMLAYTCMIENAPKFGIDV

> CmedOBP14

MFGPSFLSFATSILMVTLGSSYVNAALSVQEAAALQKEMYQFLAQCGEELGYSQEKINILSLSDLPNDACFANCFYKKIDLIKDDGKFDDDKTIEIFKRFSNDKDVLESLTLITKECAQANDANVSDGCERAKLVAECFGKREAEILPE

> CmedOBP15

MLKLVFTVFCVQALLSEVMGFKKEEVIVKMQECLGIMGLEPEDVKRGWREGDLDPCFLACFYKATKVINSDGMFDMDHTINSLKEAEILEEDKERLSKISMDCVIVNEEEVLDGVEGCERSKLLFDCIRTGLGPHQSEMSKNAGTGMW

> CmedOBP16

MAKFICLFVCVAVISSAYGVSQEEKEKFHEAIMPLLVECSEAHGVSKDDIKNAKEAGTAQGLNSCFIGCVMKKAGIIDAKGQFDVDAGVEKIKKYIANADEVAKFEEVGKTCASVNEKPVTDGEAGCDRAKLLLDCIREHKSEIPL

> CmedOBP17

MLKFFLFSVWVLGVLGDDKKEGLQMLEEEALQQAEICMEKLALDEQSIKTENGELDPCLLACIFKGFDMINSDGMFVFEAVDPKYLVGIEDKETVLKITKECASVNEEKVSDGKEGCERATLLLKCLTKLEPLLAAARKQ

> CmedOBP18

MTPFLFLCLVMAVAGRDAHNVQLSQAQKEKVHQYTAQCVKETGVKTDVLAEAKKGHFADDEALKKFTLCFFQKSGIVDSTGKLNVEAALSKLPQGVNKADATKLLEECKKKTGKDAADTAFEVFKCYSRGTKQHILA

> CmedOBP19

MFKSGICFAVVISLIKIVVSISDENKAHVQAIFVGAGEKCVTEHPISEEDSAAFKDRRFPEGENAACFSTCVLTKIGLINEQGTLSPSTALERAKEVLDSEDEIKLFQSFIDTCATEEDVKSEEKGCDLG

> CmedOBP20

MKTFVVFAVCLVAVQALTDEQKEKLKKHKGECLSETKVEEALVNKLRTGDYKTENDALKKYALCMMIKSELMTKDGKFKKDVAMAKVANPADKPQVEKLIDTCLANKGNSPHQTAWNYVKCYHEKDPKHAIFL

> CmedOBP21

MKCLKDHPVEVAELTNLQKLIVPKKMEVKCLLACAYKAEGMMTKDGKYDLEHAYKVAELTKNGDEKRLENGKKMSDLCSKVNEVEVSDGEKGCERAGLMFKCGVENAAKFGFKI

> CmedOBP22

CDVTVKGKYEVDGEILLLKLQGKGDAKFELKKAQFDYDCELVEKTGKDGLAYLSLRNSKYNYTLNDKAAIDLENLFDGNEVLGRAARELFRSYPNEIVLEIGPPMFKAIV

> CmedOBP23

AHVNIKKIMVTVDVDLEIIEKDNKKHWKVKSWRHSFELQEKSDVEFENLFPENEFLRKTTNELIASNGNDVIMEIGPLIVKAVISKIVKNVDGFFSKVPLEDLVLN

> CmedOBP24

SDVINHGKVNRELLVHQASLVNGKQSRVVRKLNSISRLCLDSIEGMSDRCQLASTYNDCLNENMLEFAFPLDIAEEAVRKMPFHLIQPNLPQEMRQSHY

> CmedOBP25

KLTPEDLKQDDMPACYKQCILKKIGLLNDAGAYDHDEVKKQMAMYTQSTDENDKLINMLDDCFEGKEQMKSDKDEEAKRI DMIFECLKNSQGL

>CmedCSP23

MKAAIAFCVFFFAALAVARPEEDRYTDRYDNIDLDEILSNKQVLVPYIKCILDQGRCTPDAKELKGHIKDALENECGKCTEAQQRGSRRVIGHLINNEPAYWTELTTKYDPEKKYSAKYEKELREIKA

>CmedCSP24

MKYLIVLCCIAVAALADDKYTDKYDNINLEEILENKRLLTAYVNCVMERGKCSPEGKELKENLQEAIETGCAKCTENQEKGAYRVIEHLIKNELDIWRELTAKYDPKGTWRKKYEERARANGITIPE

>CmedCSP25

MKSVIIMCLFGLAAVAYAKPSSTYTSKYDNINLQEITGNRRLLVPYVKCLLEQGKCTPEGKELKSHVREALAENCSKCTPAQRDGTRVVLGHLINHEPDYWNELKAKYDSEGLYSQKYEDDLRQLRH

>CmedCSP26

MKTTITLFVAFVVVLAWARPNEEMYTDRYDDINMDEILSNDKIFKSYVDCLLDKGKCTPEGKELKSKMGEALENGCAKCSEKHKGGIRRVISHLINHKESDWNALCAKYDPQRKYVDKYEKELR

>CmedCSP27

MKTFFILCVLAAAVFARPEEYTDKYDTVDLEEILSNRRLLIPYIKCGLDQGRCSSEGKELKSHIQEALENYCGKCTKAQQAGTRRVITYLINNEPEYWKQLSAKYDKDGKYAAKYEAELKTIKP

>CmedCSP28

MKLLFLAALCAVATLARAATYTDKWDNINVDEILESKRLLRGYVDCLIDKGRCTADGKTLKETIPDALENNCSKCTPKQKTGSDKVIRHLINKRPELWRELAVKYDPNNIYQEKYKDKIQEAKA

>CmedCSP29

MKCLLLVLAITIQCTLAQSATYTDRYDTINLDDVLANKRLTVAYIKCVLDKGKCTNEGKELKSHIIEALQNGCSKCTKAQREGMRRVIRHLIKYEKGYWQELVEKYDPDRVFTQKYEKELNSI

>CmedCSP30

MHSTVFLAFVTLVGFVSADLYTDRYDNINVEEILENRKLLVPYIKCVLEQGRCTPEGKELRANIKDAMQTGCTKCTDKQKKAARKVVKHLKEKEEDFYKQIVSKYDPQDKFKPTYEAFLAKED

>CmedCSP31

MKIILLTLCLGLAVLGQEQYESANDNFDISEVLTNERLLNAYAKCLLNKGPCTPEVKQVKDKLPEALATRCAKCTDKQKQMGKQLAQEVKKNHPEIWKQLVSMYDPQGKYQQAWQDFLKEN

>CmedCSP32

MRVLVVLSCLIVAVFAADKYNAKYDNFDVETLISNDRLLKAYINCFLEKGRCTPEGSDFRKTLPEAIETTCAKCTEKQKTNIRKVIKAIQAKHPKEWDALVKKNDPTGKNRDNFDKFIQG

>CmedCSP33

MKFLVVLSVVFAVVVADTYKTTHDSIDVEAVVTNPDSLKAFTGCFLDTGACNEVAASFKKVLPEATEQACAKCTPAQKHMLRRYLEEVKKTSPEDFAALNKKYDPEGKYVEALRAAIANA

>CmedCSP34

MKSYCIIFALVVAVSADFYNPKYDNFDVRPMLENDRILLSFTKCFLDEGPCTREAKDFKKAIPEALATTCGRCSPKQQQLIRAVVSAIRKKHPDSWEELIQKYDRERLYRHS

>CmedCSP35

MKYLYVLSFLLALAAADTETYSTENDNFDIDTAVADNNTLQAMAGCVLDTGDCDAVIADFKKDIPEATQTACAKCTQSQKHIFHTFLESLKNKRPADYT
