# Supplementary material for: Identification and Comparative Expression Profiles of Chemoreception Genes Revealed from Major Chemoreception Organs of the Rice Leaf Folder, Cnaphalocrocis medinalis (Lepidoptera: Pyralidae)
Source: PLoS One. 2015 Dec 11;10(12):e0144267. doi: 10.1371/journal.pone.0144267 (PMC4676629; doi:10.1371/journal.pone.0144267)
Supplement: S1 Table — (DOCX) [file pone.0144267.s003.docx]

**S1Table. Oligonucleotide primers used for expression validation analysis**

| **Gene name** | **Forward primer sequence** | **Reverse primer sequence** | **Total length** |
| --- | --- | --- | --- |
| β-Actin | CGAGCGTGGTTACTCATTCA | ATGACTTCTCGAGCGAGCTG | 137bp |
| CmedOrco | TGAGCTGTTCCGAGCTTCGT | CAGGGTCATGCCGAAGTCCT | 109bp |
| CmedCSP6 | CTGAGGGGCTACGTTGACTG | ATGCCTGATGACCTTGTCGG | 150bp |
| CmedCSP16 | CGACGAGTCCACGTACACCA | ACGTAGCTGGTGAGAAGGCG | 84bp |
